# Potassium Salts of 9‑Oxo‑9*H*‑fluoren-4-carboxylic Acid: Phase Formation, Crystal Structure, and Electrochemical Behavior in Potassium-Ion Batteries

**DOI:** 10.1021/acs.chemmater.6c01146

**Published:** 2026-09-10

**Authors:** Irene Gómez-Berenguer, Patricia Delgado-Martínez, Álvaro Lobato, Bernardo Herradón, Elizabeth Castillo-Martínez

**Affiliations:** † Departamento de Química Inorgánica, 16734 Universidad Complutense de Madrid, 28040 Madrid, Spain; ‡ Instituto de Química Orgánica General, Consejo Superior de Investigaciones Científicas, 28006 Madrid, Spain; § Unidad de Difracción de Rayos X, Centro de Asistencia a la Investigación de Técnicas Químicas, Universidad Complutense de Madrid, 28040 Madrid, Spain; ∥ Departamento de Química Física, Universidad Complutense de Madrid, 28040 Madrid, Spain

## Abstract

Potassium-ion batteries (KIBs) are attracting increasing interest as alternatives to lithium-based systems owing to the natural abundance and low cost of potassium. Organic carbonyl compounds have emerged as promising electroactive materials, as their redox properties can be tuned through molecular design and crystal structure. Here, we examine potassium derivatives of 9-oxo-9*H*-fluoren-4-carboxylic acid (HFl). Depending on reaction conditions, two distinct crystalline phases are obtained from the parent acid: the fully deprotonated potassium salt (KFl) and a hydrogen potassium carboxylate (HKFl_2_). While acid salts of monoprotic carboxylic acids are well-known, their formation and structural implications in fluorenone-based systems remain largely unexplored. Single-crystal X-ray diffraction reveals that both structures comprise extended K–O coordination networks built from distorted KO_6_ environments, while HKFl_2_ additionally features strong O–H···O^–^ hydrogen bonds linking neutral and deprotonated molecules. KFl electrodes show reversible K-ion insertion at ca. 1.5 V vs K^+^/K, despite pronounced first-cycle irreversibility and capacity fading, which may be related to electrolyte-dependent side processes and active-material dissolution. Ex situ diffraction shows that KFl is the electrochemically active phase, whereas HKFl_2_ converts into KFl upon electrolyte contact. These results demonstrate that subtle variations in protonation state lead to distinct crystalline phases that directly govern electrochemical behavior.

## Graphical abstract

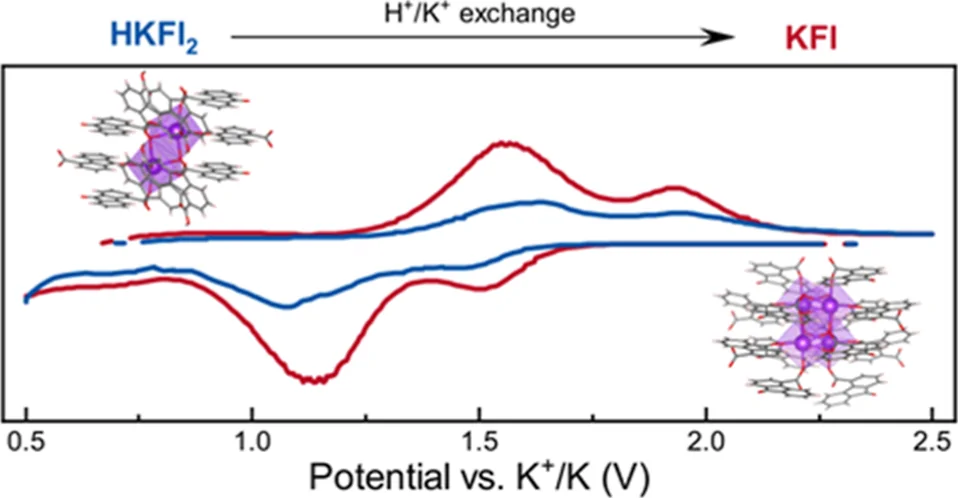

## Introduction

The development of efficient and sustainable electrode materials remains a key challenge for next-generation potassium-ion batteries (KIBs), which are being actively explored as an alternative to lithium-ion batteries (LIBs) for large-scale and stationary energy storage applications. Potassium is highly abundant in the earth’s crust (2.09 wt %, compared to 0.0017 wt % for lithium) and widely distributed. In addition, the reversible intercalation of K^+^ into graphite^1^ and the possibility of using aluminum current collectors enhance the industrial appeal of KIBs. Nevertheless, the larger ionic radius of K^+^ imposes stringent structural requirements on electrode materials, and the limited availability of robust hosts capable of reversible K^+^ storage remains a major obstacle to the practical development of KIBs.^2–5^

Organic electrode materials have been broadly investigated across alkali-ion battery technologies owing to their reliance on earth-abundant elements, structural diversity, and molecular-level tunability of redox activity. In contrast to inorganic electrodes, organic materials typically store charge through reversible molecular redox reactions rather than lattice-based intercalation processes, enabling flexible molecular design and mitigating long-range structural strain during cycling. However, organic electrodes are also commonly limited by their intrinsically low electronic conductivity, lower energy density and, in many cases, by partial solubility in conventional electrolytes, which can lead to loss of active material and capacity fading. As a result, achieving an appropriate balance between molecular redox activity, electronic conductivity within the organic solid, and solid-state stability remains a central challenge in the development of organic electrode materials for KIBs.

Among the various classes of organic electrode materials, carbonyl-based aromatic compounds have been extensively investigated due to their reversible redox activity and compatibility with alkali-ion storage mechanisms. These materials predominantly operate as n-type electrodes, with charge storage governed by the reversible reduction of carbonyl functionalities, along with the coordination of alkali metal cations, providing flexibility to modulate the redox behavior through changes in molecular structure.^4–7^ Within this broad class of carbonyl-based n-type organic electrode materials—including molecular systems such as quinones, aromatic ketones, imides, and anhydrides—derivatives of 9-oxo-9*H*-fluorene (also known as fluoren-9-one) represent a structurally simple subclass that combines a rigid aromatic framework with a redox-active carbonyl group.

Derivatives of fluoren-9-one are well-established molecular scaffolds in organic and materials chemistry, owing to their rigid polycyclic aromatic framework and chemically robust carbonyl functionality. Their distinctive photophysical and electron-accepting properties have enabled widespread use in sensing, photoactive and optoelectronic materials, as well as in multifunctional supramolecular and hybrid systems. In addition, their reversible redox behavior has motivated occasional applications in electrochemical systems.^8–14^ In the context of electrochemical energy storage, fluoren-9-one derivatives have been extensively investigated as redox-active species in organic redox-flow batteries, where they have demonstrated reversible redox behavior and electrochemical stability.^15–17^ Beyond redox-flow batteries, these compounds have only sporadically been explored in other battery-related roles, including early studies as cathode materials in lithium metal batteries,^18^ redox-active components in hybrid carbon-organic electrodes,^19^ and as electrolyte additives, where fluoren-9-one has been shown to aid uniform sodium deposition and prolonged cycling stability in sodium-metal batteries.^20^ However, the use of fluoren-9-one-based derivatives as structurally defined solid-state electrode materials in metal-ion batteries—particularly in potassium-based systems—remains largely unexplored.

In this work, we investigate potassium derivatives of a fluoren-9-one based carboxylate (Scheme 1) as organic solid-state materials, within the context of potassium-ion battery research. Emphasis is placed on understanding how protonation state and synthetic conditions govern phase formation and solid-state structure, and how these factors ultimately determine the electrochemical behavior.

**Scheme 1:**
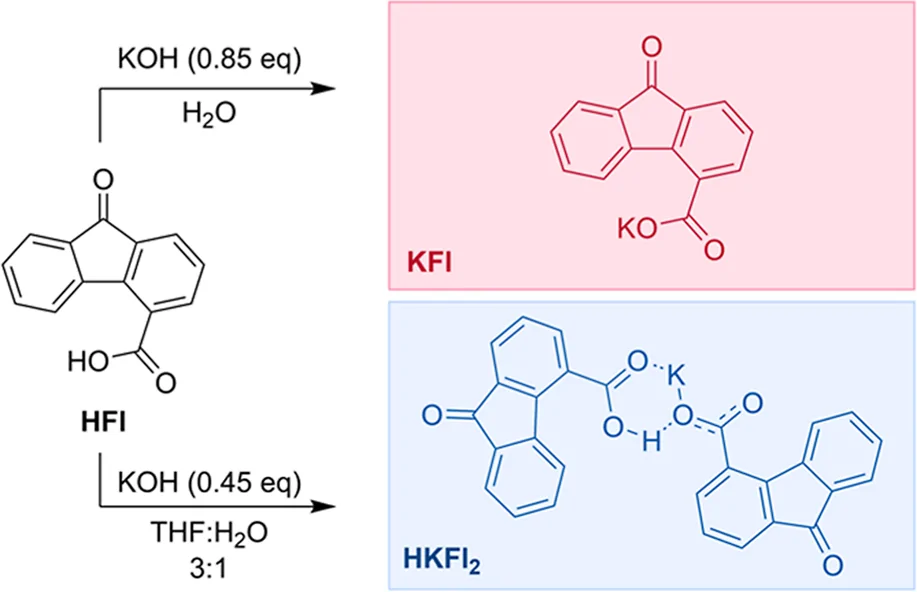
Synthesis of KFl and HKFl_2_

## Results and Discussion

### Synthesis and Characterization

All products were crystalline yellow powders. Depending on the reaction conditions, two different pure products and a mixture of them were obtained from the reaction of 9-oxo-9*H*-fluoren-4-carboxylic acid (HFl) and KOH: the fully deprotonated potassium salt (KFl), the hydrogen potassium carboxylate (HKFl_2_)—containing one carboxylate unit per neutral acid molecule—, and a mixture of both (Scheme 1). This type of phase is consistent with acid salts of carboxylic acids, which are commonly observed, and whose formation is strongly influenced by reaction conditions such as stoichiometry and solvent, as observed here.^21^

Performing the acid–base reaction in water, using a 1:0.85 molar ratio of HFl/KOH led exclusively to KFl. Under these conditions, the acid is poorly soluble whereas KOH readily dissolves, leading to a heterogeneous reaction in which the potassium salt forms in solution. The excess unreacted acid is removed by filtration, and the product is subsequently isolated by solvent evaporation.

In contrast, in a THF/H_2_O (3:1 v/v) mixture, both reagents and products were soluble, and the relative amount of base became decisive: a 1:0.45 molar ratio of HFl/KOH afforded HKFl_2_, whereas an intermediate 1:0.70 ratio yielded a mixture of KFl and HKFl_2_. In this case, solvent evaporation occurs prior to phase separation, promoting coprecipitation of protonated and deprotonated species and favoring the formation of the acid salt phase. Subsequent washing with ethyl acetate removed excess acid but did not alter the composition of the other precipitated phase/s.

These contrasting solubility behaviors and isolation sequences—precipitation from solution in water vs coprecipitation followed by washing in THF/H_2_O—create distinct reaction environments (heterogeneous vs homogeneous), which likely determine the preferential formation of each phase. Overall, these observations highlight the key role of acid–base stoichiometry, solvent polarity and isolation conditions in directing the synthetic outcome. Although KFl and HKFl_2_ are distinct compounds, they will hereafter be referred to as phases for simplicity.

The three isolated solids were further studied by PXRD, TGA, IR and NMR spectroscopy to distinguish the different phases obtained, while SEM was used to compare their morphology.

PXRD analysis (Figure 1a) clearly shows the formation of two distinct crystalline phases. HKFl_2_ is not a physical mixture of HFl and KFl, but an independent crystalline phase with its own diffraction pattern. In contrast, the phase mixture shows a pattern that can be assigned to a superposition of the reflections of KFl and HKFl_2_, with no evidence of additional crystalline phases. These results confirm that the synthetic conditions control the formation of either the fully deprotonated potassium salt, the hydrogen potassium carboxylate phase, or a mixture of both.

**Figure 1:**
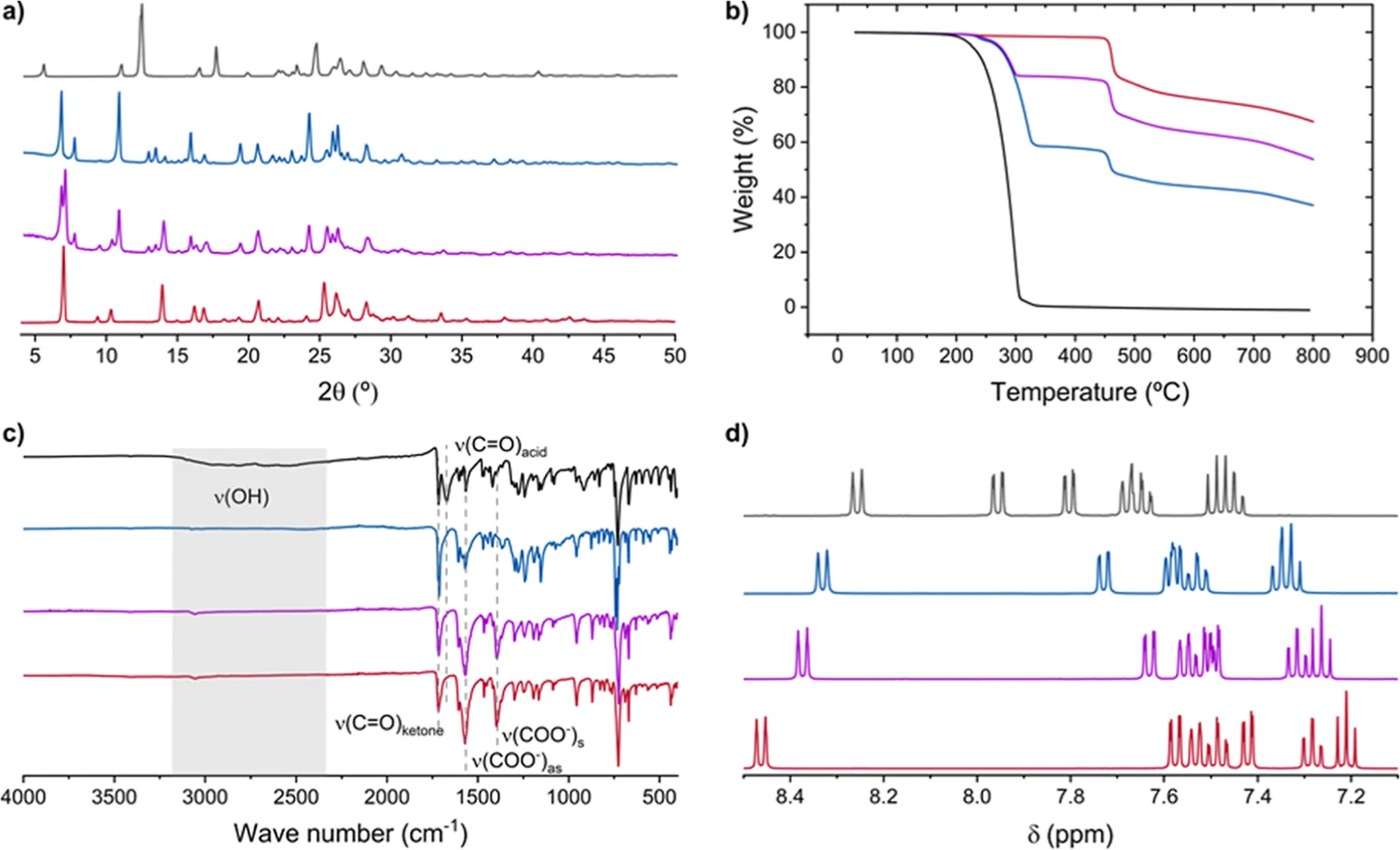
(a) PXRD, (b) TGA, (c) IR spectra, (d) ^1^H NMR (DMSO-*d*_6_). Black line HFl, blue line HKFl_2_, violet line KFl + HKFl_2_, red line KFl.

Thermogravimetric analysis further supports the formation of distinct phases (Figure 1b, Table S1). HFl decomposes in a single step at relatively low temperature (*T*_p_ = 298 °C), whereas KFl is more stable, decomposing at *T*_p_ = 462 °C and leaving a potassium-rich residue. On the other hand, HKFl_2_ and the phase mixture display two-step profiles consistent with sequential acid release and salt decomposition. The first step in HKFl_2_ occurs at a higher temperature than in the free acid (312 °C vs 298 °C), reflecting stabilization by ionic interactions and hydrogen bonding, while the second one occurs at temperatures slightly below that of the pure salt (458 °C vs 462 °C), consistent with a less cohesive lattice.

Infrared spectroscopy (Figure 1c) clearly differentiates HFl from its potassium salt derivatives. The acid exhibits a broad O–H stretching band (3200–2200 cm^–1^) and a carboxylic ν­(C=O) vibration at 1673 cm^–1^, both absent after deprotonation. The ketone ν­(C=O) band persists at ∼1715 cm^–1^ in all phases. In KFl and in the phase mixture, intense carboxylate ν_as_/ν_s_(COO^–^) bands appear at ∼1570/1395 cm^–1^, whereas in HKFl_2_ only the ν_as_(COO^–^) band is clearly observed, together with a residual O–H bending absorption at 921 cm^–1^.^22–24^ The absence of the ν_s_(COO^–^) band in HKFl_2_ can be attributed to the hydrogen-bonding arrangement, which may reduce the dipole moment change of the symmetric stretching mode and render it very weak and difficult to detect.^25,26^

In the ^1^H NMR spectra recorded in DMSO-*d*_6_ (Figure 1d), HFl displays a broad resonance at 13.67 ppm (Figure S1) attributable to the carboxylic OH, which is absent in the three derived solids. In the aromatic region, all resonances undergo noticeable chemical-shift variations among the different samples. In particular, the aromatic signal at lower field (δ > 8 ppm) becomes further deshielded upon deprotonation, whereas the remaining aromatic resonances are shifted slightly upfield. This most downfield signal is tentatively assigned to H-3, located ortho to the carboxyl group at C-4 (Figure 2).

**Figure 2:**
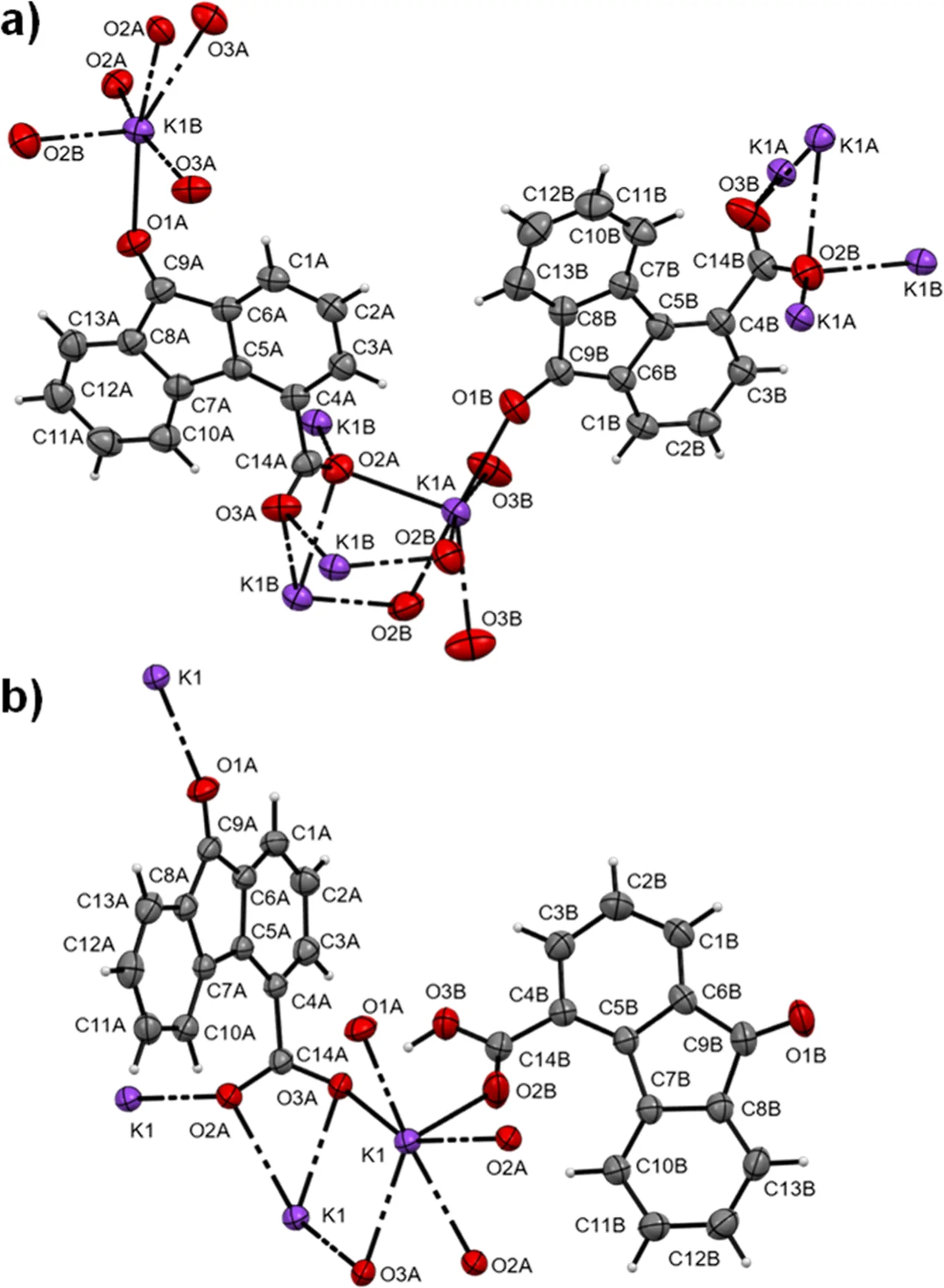
A view of the molecular structures of (a) KFl (CCDC 2380849) and (b) HKFl_2_ (CCDC 2345911). Displacement ellipsoids enclose 30% probability level. The images were generated using *Mercury* (CCDC).^29^

The differences observed in Figure 1d suggest changes in the electronic environment of the fluoren-9-one core. These may be related to the different protonation states in solution and to interactions with DMSO-*d*_6_. In this solvent, fast proton exchange between HFl and FlCO_2_^–^ is expected, leading to averaged NMR signals.^27^ As illustrated in Figure S2, repeated ^1^H NMR measurements of the same phase-mixture sample show small variations in chemical shifts, likely associated with subtle changes in the solution environment, such as differences in trace water content in DMSO-*d*_6_. Consequently, the chemical shifts cannot be reliably used to determine the relative proportion of protonated and deprotonated species in solution.

Additional solvent-dependent effects were observed when comparing spectra recorded in DMSO-*d*_6_, D_2_O and acetone-*d*_6_ (Figures S3 and S4). The free acid and its potassium carboxylate exhibit opposite solvent-dependent shifts of the most downfield aromatic signal, reflecting different interaction modes with the solvent: hydrogen bonding in the acid versus electrostatic and coordination effects in the salt. In the ^13^C NMR spectra recorded in DMSO-*d*_6_ (Figure S5), only minor differences are observed, with more closely grouped aromatic signals, consistent with the lower sensitivity of ^13^C chemical shifts to solvent effects.

SEM analysis (Figure S6) shows elongated, lamellar crystals for all samples, with only minor morphological variations among the phases. HKFl_2_ exhibits more defined rod-like crystallites, the phase mixture shows slight surface inhomogeneity, and KFl forms thicker, more compact layered crystals. Overall, the morphology is largely preserved across the series.

### Single-Crystal X-ray Diffraction Analysis

Single crystals of KFl and HKFl_2_ were grown by slow recrystallization from EtOH and characterized by single-crystal X-ray diffraction. Crystals of KFl were obtained from the pure salt, while HKFl_2_ crystals were isolated by recrystallization of the phase mixture under identical conditions. Table 1 summarizes the crystallographic parameters of KFl and HKFl_2_, together with those of HFl reported in the literature.^28^

**Table 1:** Selected Crystallographic Parameters of HFl, HKFl_2_ and KFl^a^ ^,^ ^b^

	HFl	HKFl_2_	KFl
formula	C_14_H_8_O_3_	C_28_H_15_KO_6_	C_28_H_14_K_2_O_6_
*M* (g/mol)	224.21	486.50	524.59
crystal system	monoclinic	triclinic	monoclinic
space group	*P*2_1_/*c*	*P*1̅	*P*2_1_/*c*
*a*/Å	3.843(3)	6.9723(2)	17.2425(4)
*b*/Å	7.986(5)	11.8536(4)	18.9409(5)
*c*/Å	32.69(2)	13.4853(5)	7.07880(10)
α/deg	90	82.065(2)	90
β/deg	96.64(4)	84.593(2)	94.143(2)
γ/deg	90	84.943(2)	90
*Z*	4	2	4
*Z*′	1	1	1
*V* (Å^3^)	996.553	1095.74	2305.81
ρ_calc_ (g/cm^3^)	1.50	1.475	1.511
*R*-factor (%)	6.8	5.64	6.28

a Common details: radiation Cu K_α_ (λ = 1.54178).

b Crystal data for HFl were taken from the literature.^28^ Full refinement details for HKFl_2_ and KFl are recorded in Table S2.

HFl crystallizes in a monoclinic unit cell, with space group (S.G.) *P*2_1_/*c,* stabilized by O–H···O and formation of HFl dimers. On the other hand, HKFl_2_ crystallizes in a system with lower symmetry—triclinic in S.G. *P*1̅. These differences are consistent with the coexistence of protonated and deprotonated carboxyl groups, corresponding to an acid salt of a carboxylic acid. For comparison, the unit-cell volume can be normalized per fluorenecarboxylate unit (4 in HFl, 4 in HKFl_2_ and 8 in KFl). On this basis, a ∼10% volume increase is observed from HFl to HKFl_2_, consistent with the larger ionic size of K^+^ compared to H^+^, whereas a smaller increase of ∼5% is observed from HKFl_2_ to KFl, indicating that the structural expansion is significantly more pronounced during the initial proton–cation exchange step than upon complete substitution. Similar to the parent acid, KFl crystallizes in a monoclinic system but exhibits an expanded unit cell relative to HFl, reflecting the replacement of hydrogen-bonded dimers by extended K–O coordination networks. The smaller expansion observed upon full substitution suggests that part of the structural reorganization required to accommodate K^+^ ions is already achieved in the HKFl_2_ phase.

Figure 2 displays the ORTEP representations of KFl and HKFl_2_. Although *Z*′ = 1 for both structures, two distinct local coordination environments are observed. In HKFl_2_ this nonequivalence has a clear origin: the keto group of the carboxylate links to K^+^, whereas the oxo functionality of the neutral acid remains nonbonded (Figure 2b). Each potassium cation is 6-fold coordinated by oxygen atoms from carboxylate and carbonyl groups, forming distorted KO_6_ octahedra that govern the three-dimensional packing. In contrast, in KFl, all carboxylate and keto groups are involved in K···O coordination without any dangling keto group, and again each potassium cation adopts a distorted 6-fold oxygen environment (Figure 2a). Subtle variations in coordination geometry give rise to distinct local environments within the extended network.

In addition to the dominant role of K···O coordination, hydrogen bonding also makes a significant contribution in directing the supramolecular organization. Whereas the reported HFl structure features the classical R_2_^2^(8) carboxylic-acid dimer,^30^ the coexistence of neutral and deprotonated species in HKFl_2_ gives rise to an R_1_^1^(6) hydrogen-bond motif (O3B–H­(O3B)–O1A–K1–O2B–C14B) (Figure 2b) that specifically links COOH and COO^–^ groups. This motif corresponds to a type B acid salt according to the Speakman classification, characterized by an asymmetric O–H···O^–^ interaction between distinct COOH and COO^–^ groups.^21^ This hydrogen bond stabilizes the structure and, together with the KO_6_ coordination units, plays a decisive role in defining the distinct local environments and overall three-dimensional packing (Figure 3). In KFl, the absence of acidic protons eliminates such specific hydrogen-bond interactions, leaving K···O coordination as the primary structure-directing force. The fluoren-9-one cores remain nearly planar across all solids (Figure 3). The angle between the mean planes of the two benzenoid rings (∼4°) corresponds to a slight butterfly distortion, which is only modestly larger than that reported for unsubstituted fluoren-9-one (∼1°),^31^ indicating that neither hydrogen bonding nor K–O coordination induces significant additional perturbation of the fluorene backbone planarity.

**Figure 3:**
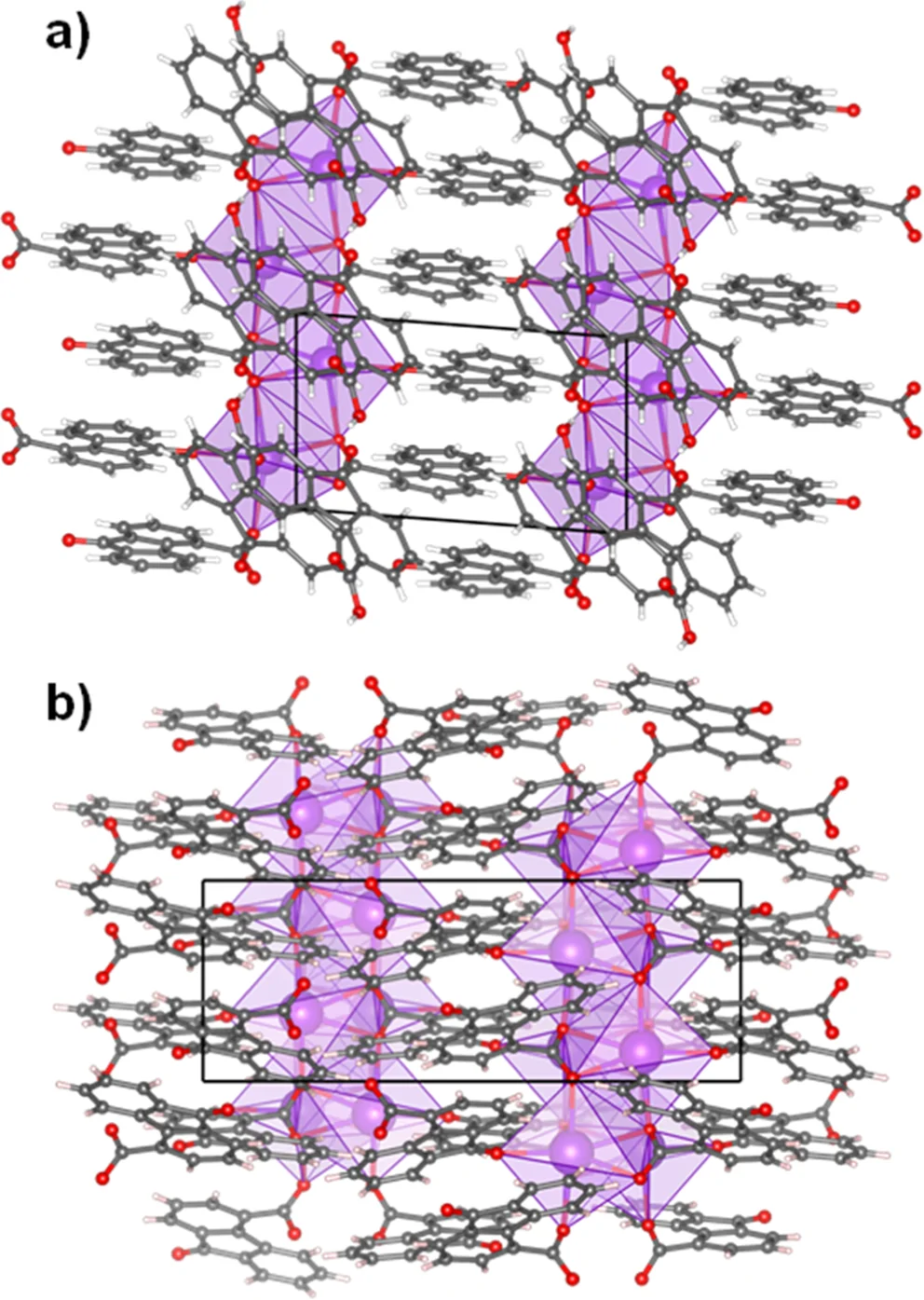
Crystal structure of HKFl_2_ (CCDC 2345911) viewed along the *c*-axis (a) and of KFl (CCDC 2380849) viewed along the *a*-axis (b). The images were generated using *VESTA*.^32^

Table 2 summarizes selected bond metrics for the acid and the two phases obtained. The carbonyl C=O distances of the ketone functionality remain nearly identical in all structures (1.216–1.222 Å). In HKFl_2_, no significant difference is observed between the coordinated and noncoordinated carbonyl groups, indicating that K···O interactions only weakly perturb the geometrical parameters of this functionality. In contrast, the carboxyl region shows the expected evolution from COOH to COO^–^, with redistribution of C–O/C=O bond lengths and enlarged O–C–O angles, consistent with increasing delocalization upon deprotonation. Hydrogen-bond parameters further differentiate HFl and HKFl_2_: the typical carboxylic-acid hydrogen bond present in HFl is replaced in HKFl_2_ by a shorter O···O distance (2.567 Å vs 2.642 Å in HFl), a shorter H···O contact (1.654 Å vs 1.682 Å) and a nearly linear O–H···O angle (174.76° vs 158.04°), indicating a significantly stronger interaction. This is consistent with the behavior of acid salts of carboxylic acids, which are known to exhibit very short O···H···O hydrogen bonds (∼2.44–2.50 Å) associated with strong hydrogen-bonding interactions between coexisting COOH and COO^–^ groups.^21^ Full structural data are provided in the Supporting Information.

**Table 2:** Selected Coordination and Hydrogen-Bond Parameters^a^ ^,^ ^b^

parameter	HFl	HKFl_2_ (A)	HKFl_2_ (B)	KFl (A)	KFl (B)
K···O Coordination					
K···O_carbonyl_ (Å)	-	2.709		2.694	2.695
K···O_carboxylate_ (Å)	-	2.709–2.945	2.651	2.670–2.875	2.717–2.906
Ketone Region					
C=O_carbonyl_ (Å)	1.216	1.219	1.222	1.217	1.219
COO^–^/COOH Geometry					
C=O_acid_ (Å)	1.241	-	1.214	-	-
C–O_carboxylate_ (Å)	-	1.245	-	1.229/1.261	1.233/1.244
O–C–O (deg)	121.66	123.66	122.82	126.14	125.41
C–OH (Å)	1.286	1.304		-	
Hydrogen Bonding Geometry					
O–H (Å)	1.005	0.916		-	
H···O (Å)	1.682	1.654		-	
O···O (Å)	2.642	2.567		-	
O–H···O (deg)	158.04	174.76		-	

a Values correspond to the two nonequivalent molecular environments (A and B) in the asymmetric unit.

b CCDC deposition numbers: 2345911 for HKFl_2_ and 2380849 for KFl.

Figure 3 illustrates the molecular packing arrangements in the HKFl_2_ and KFl phases. The KO_6_ distorted octahedra described above connect through edge sharing oxygen atoms to form 1D zigzag chains parallel to the *a* axis in HKFl_2_. In KFl, the octahedra form 1D zigzag double chains connected through corner sharing oxygen atoms, running parallel to the *c* axis. The organic moieties fill the interchain regions forming layers. In HKFl_2_, only the carboxylate-bearing molecules participate in π–π stacking (interplanar angle = 3.7°, centroid–centroid distance = 3.82 Å, slippage = 1.53 Å), whereas the neutral acid molecules remain nonstacked, giving rise to alternating columns of stacked and unstacked units. In KFl, in contrast, both independent molecules participate in π stacking with nearly parallel orientations (interplanar angle = 0°), with centroid–centroid separations of 3.61 and 3.75 Å and slippages of 1.51 and 1.62 Å for asymmetric molecules A and B, respectively. The stacking motif also differs: while in the free acid the ketone groups overlap, in the salts the overlap alternates between ketone and carboxylate groups, producing a repeating ketone–carboxylate–ketone sequence along the stacking axis. The projections in Figure 3 emphasize the similarity of these stacking motifs and of the [KO_6_] octahedra zigzag chains, across the two phases, despite their different symmetry.

In addition to π–π stacking and hydrogen bonding, both HFl and HKFl_2_ display C–H···π interactions involving the H-3 proton (Figure S7). In the free acid, H-3 interacts laterally with the π system of a neighboring ring as a result of the parallel, offset stacking of fluorenone units. In the HKFl_2_ phase, by contrast, the H-3 proton of the carboxylate approaches more directly the π system of a neutral acid fragment, adopting a nearly perpendicular geometry. No C–H···π interactions are detected in the fully deprotonated potassium salt, where the packing is instead dominated by extended K–O coordination and regular π–π stacking.

### Solid-State Nuclear Magnetic Resonance Spectroscopy

In order to complement the crystallographic characterization of the phases, we have performed studies by solid-state nuclear magnetic resonance spectroscopy (ssNMR) under magic-angles spinning (MAS) conditions. This technique is particularly suitable for distinguishing between different crystalline phases and probing the local chemical environment of nuclei, even in samples that are not suitable for single-crystal X-ray diffraction. ssNMR offers an accessible and versatile approach, enabling the structural analysis of the three materials under study and the starting acid.^33,34^

Figure 4a shows the solid-state ^1^H MAS NMR spectra of the starting acid and the three prepared materials. In HFl, the COOH proton resonates at 13.6 ppm, whereas in HKFl_2_ a residual COOH signal is shifted downfield to 15.0 ppm, which is also visible, with lower intensity, in the phase mixture, as expected. In KFl, only the aromatic resonance at ∼7.0 ppm remains. The pronounced downfield shift of the COOH proton in the HKFl_2_ phase is indicative of stronger hydrogen bonding within its lattice. Although this shift is substantial, it remains smaller than that reported for partially potassiated hybrid carboxylate/Schiff-base systems, where ^1^H resonances as far downfield as 20 ppm have been observed; in those materials, however, the H bond distance and the degree of potassiation were not unambiguously established.^35^

**Figure 4:**
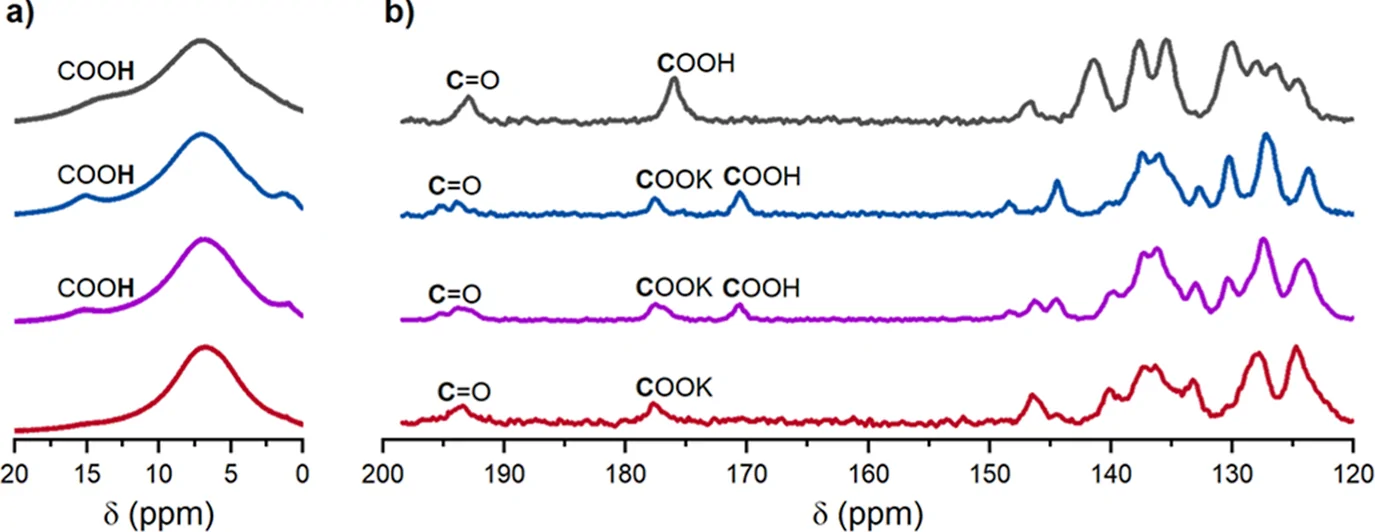
(a) Solid-state ^1^H MAS NMR spectra, (b) solid-state ^13^C MAS NMR spectra. Black line HFl, blue line HKFl_2_, violet line KFl + HKFl_2_, red line KFl.

Figure 4b shows the solid-state ^13^C MAS NMR spectra. In both HKFl_2_ and the phase mixture, two distinct resonances are observed in the carboxyl region: a signal at 177.6 ppm corresponding to the carboxylate anion (COO^–^), identical to that observed in KFl, and a COOH signal at 170.6 ppm, shifted upfield relative to the free acid (176.0 ppm). This upfield displacement reflects the different hydrogen-bonding environment in HKFl_2_. Instead of forming the classical R_2_^2^(8) acid dimer, the COOH group is involved in a strong O–H···O^–^ interaction with a neighboring carboxylate, which modifies the electron distribution within the carboxyl group and leads to increased shielding of the carboxyl carbon. These phases also exhibit two nonequivalent ketone resonances, reflecting coordination to K^+^, in agreement with their crystallographic asymmetry. The aromatic region further differentiates the phases, with splitting and additional signals in HKFl_2_ and in the phase mixture, consistent with the coexistence of protonated and deprotonated units. Taken together, the ssNMR data corroborate the crystallographic findings and provide direct evidence of phase coexistence in the mixed material. This was not the case in solution NMR (Figure 1d), which suggested a unique phase of intermediate H^+^/K^+^ composition.

### Phases Stability through DFT Calculations

The relative energies of the HKFl_2_ and KFl phases were evaluated via density functional theory (DFT) calculations by considering Reaction R1

R1 $$K_{8}C_{112}O_{24}H_{56}\left(FlCO_{2}K\right)+2C_{56}O_{12}H_{32}\left(FlCO_{2}H\right)\to 4K_{2}C_{56}O_{12}H_{30}\left({\left(FlCO_{2}\right)}_{2}HK\right)$$

The energies of the individual phases were obtained by optimizing the experimental unit cells of HFl,^28^ HKFl_2_ and KFl. At 0 K and under static conditions, the calculated Gibbs energy difference for the reaction is −4.43 kcal·mol^–1^. While this value does not directly account for solvation or finite-temperature effects present in solution as in the experiments, it indicates a thermodynamic preference for the formation of the HKFl_2_ phase in the solid state. This trend is consistent with previous reports on acid salts of carboxylic acids, which are often found as stable phases and can, under suitable conditions, be preferentially obtained over fully deprotonated salts.^21^

### Electrochemical Performance

To evaluate the potential of the potassiated phases derived from 9-oxo-9*H*-fluoren-4-carboxylic acid as electrode materials for KIBs, galvanostatic charge–discharge (GCD) tests were performed in half-cell configuration within the voltage range 0.5–2.5 V vs K^+^/K. HFl was not evaluated electrochemically because preliminary solubility tests showed complete dissolution in the electrolyte solvents examined. This makes it unsuitable as a solid-state electrode material under these conditions.

Five electrolytes were examined for KFl: 2.5 M potassium bis­(fluorosulfonyl)­imide (KFSI) in triethyl phosphate (TEP), 3.9 M KFSI in dimethoxyethane (DME), 1 M KFSI in 1:1 (wt) ethylene carbonate/diethyl carbonate (EC/DEC), 1 M KFSI in 2-methyltetrahydrofuran (MeTHF) and 1 M KPF_6_ in triethyl phosphate (TEP) (Figure S9). The electrolyte was varied because the solvent, potassium salt and salt concentration can all affect the electrochemical response of organic electrodes, by influencing K^+^ solvation, active-material dissolution, ion transport and interfacial stability. Among the electrolytes tested, 2.5 M KFSI in TEP gave the most defined electrochemical response (Figure S9a) and was therefore selected for a more detailed electrochemical study. This electrolyte also benefits from the use of TEP, which has a high boiling point and flame-retardant properties.^36,37^

In the other electrolytes, KFl also showed a high capacity during the first discharge, as also observed in 2.5 M KFSI in TEP, but this was followed by severe capacity fading. The most pronounced case was observed in 3.9 M KFSI/DME (Figure S9c): after a first discharge close to 400 mAh/g, the following charge delivered less than 10 mAh/g. Although DME-based concentrated electrolytes have been widely used in KIBs half-cell studies,^37^ mainly because they enable stable K metal plating and stripping,^38^ these results show that their performance strongly depends on the electrode material.

To better understand the strong capacity fading observed in the different electrolytes, qualitative solubility tests were carried out by placing 1 mg of KFl in a vial in contact with 0.5 mL of each electrolyte for 48 h (Figure S10). After this time, a yellow coloration was observed in all of the solutions, indicating partial dissolution of KFl. The coloration was more intense for 3.9 M KFSI in DME and 1 M KFSI in EC/DEC, suggesting a higher apparent solubility in these electrolytes. In 2.5 M KFSI in TEP, some coloration was also observed, indicating that dissolution cannot be completely avoided. These results suggest that active-material dissolution is likely an important contributor to the capacity fading observed during cycling. Since partial dissolution occurs upon simple contact with the electrolyte, some active material may already be dissolved before cycling starts. This could also contribute to the low Coulombic efficiency, either by reducing the amount of material available for reversible charge storage or by undergoing reduction reactions at the potassium metal counter electrode. Additional contributions from reduced soluble species, irreversible electrochemical processes and structural rearrangements may also be involved.

The pronounced first-cycle irreversibility was further examined by comparing the response of KFl with that of a C65 blank electrode measured in 2.5 M KFSI in TEP (Figure S11). The blank electrode shows a large irreversible reduction during the first discharge, suggesting that the irreversibility observed for KFl is not exclusively associated with the active material, but also with parasitic processes involving the conductive carbon, the electrolyte and/or the K metal counter electrode. Nevertheless, the first plateau of KFl starts at around 1.4 V, whereas the main reduction of the C65 blank appears at a lower potential, close to 1.25 V. This difference suggests that the higher-potential process is mainly related to KFl reduction. Therefore, the electrolyte strongly affects the practical shape of the first-discharge profile through solubility, interfacial stability, polarization and side reactions, but the active material remains responsible for the main redox contribution.

A similar irreversible contribution has also been reported for a C65 electrode in 3.9 M KFSI in DME under comparable conditions,^35^ although the comparison is limited to the common potential range because the reported cell was discharged down to 0.35 V. This supports that electrolyte/carbon/K-metal side processes can also contribute to the first-discharge profile in the DME-based electrolyte. However, as in TEP, the main first-discharge plateau of KFl appears at a higher potential than the C65 response, indicating that the active material also contributes to this process.

The galvanostatic charge–discharge curves of the three materials in 2.5 M KFSI in TEP are shown in Figure 5a. All three electrodes exhibit a pronounced first-cycle irreversible loss, with low initial Coulombic efficiencies of 31.3% for KFl, 7.9% for HKFl_2_ and 25.1% for the mixed-phase sample. Among them, KFl delivers the best performance, reaching a discharge capacity of 151 mAh/g in the second cycle, which corresponds to the insertion of approximately 1.5 K^+^ ions per formula unit. However, the Coulombic efficiency in this cycle remains low (67.4%), in agreement with the irreversible and dissolution-related contributions discussed above. The capacity continues to decline with cycling, giving a discharge capacity of 68 mAh/g and a Coulombic efficiency of 95.9% at cycle 20. In contrast, the mixture of phases delivers lower capacities, while the HKFl_2_ exhibits the poorest performance, further confirming the superior electrochemical behavior of KFl.

**Figure 5:**
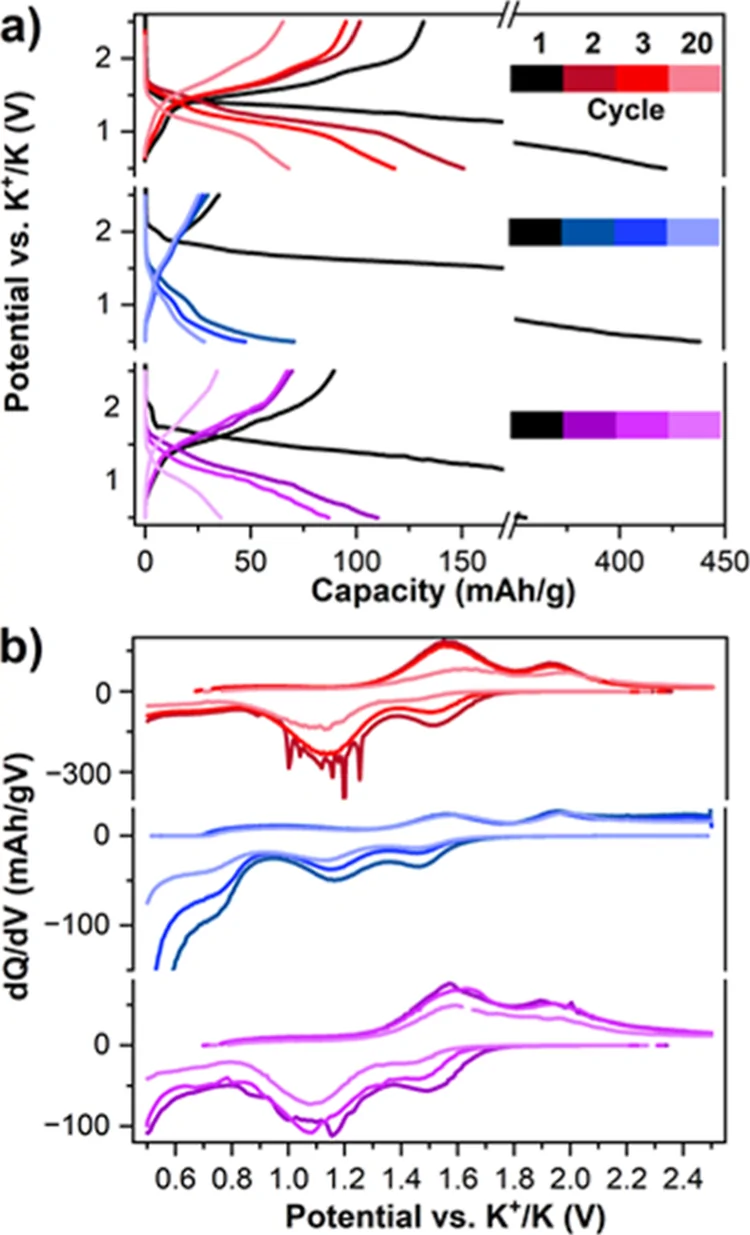
Electrochemical performance of the three materials. (a) Galvanostatic voltage charge and discharge and (b) d*Q*/d*V* plots. The d*Q*/d*V* plots are shown with different *y*-axis scales to improve visualization. Red line KFl, blue line HKFl_2_, violet line KFl + HKFl_2_.

The three materials display comparable redox behavior, as shown in Figure 5b, with two reduction peaks at ∼1.5 V and ∼1.1 V and two oxidation peaks at ∼1.6 V and ∼2.0 V. The higher-potential reduction process is tentatively associated with the ketone carbonyl group of the fluoren-9-one core, which is expected to be more readily reduced than the carboxylate group.^4^ The lower-potential process may involve further reduction and/or additional interactions involving carboxylate groups. In HKFl_2_, additional redox features appear below 0.9 V, overlapping with the low-potential response observed for the C65 blank electrode (Figure S11). These features are more evident for HKFl_2_ because its electrochemical response is weaker and less defined than that of KFl (note the different scale), rather than due to an intrinsically higher activity of C65 in this electrode. Similar, although less intense, contributions are also observed in the mixed-phase sample.

The similarities in the voltage profiles of the three samples can be rationalized by PXRD analysis. As shown in Figure 6a, a comparison between the diffractogram of pristine HKFl_2_ and that recorded after 24 h in contact with the electrolyte reveals the emergence of reflections characteristic of KFl, indicating that HKFl_2_ spontaneously converts into KFl upon exposure to the KFSI-containing electrolyte. This transformation is likely accompanied by a proton–potassium exchange process involving the electrolyte. Protonation of the FSI^–^ anion, leading to the possible formation of HFSI, could be involved, although this species was not directly detected. This behavior can be related to the acid–carboxylate nature of HKFl_2_. Although the O–H···O^–^ hydrogen bond stabilizes this arrangement in the crystalline solid, the presence of an acidic proton makes the phase chemically sensitive to the KFSI-containing electrolyte. As a result, HKFl_2_ does not behave as an independent electrochemically active phase under these conditions, but rather converts into KFl upon electrolyte contact.

**Figure 6:**
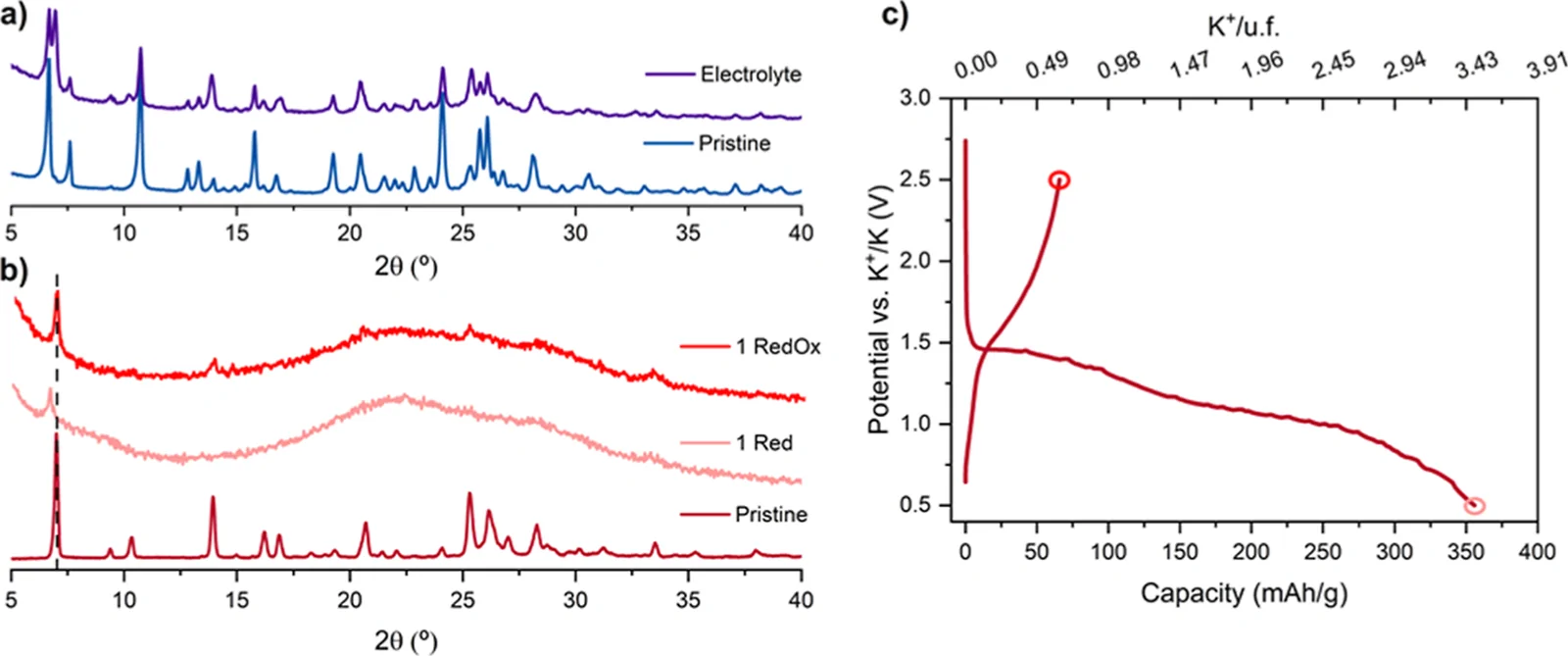
PXRD patterns of (a) HKFl_2_ in the pristine state and after 24 h in contact with the electrolyte, and (b) KFl in the pristine state, after the first reduction, and after the subsequent reoxidation. (c) GCD curve of the first cycle, with the points corresponding to the PXRD measurements of KFl in (b) indicated as circles.

The observed transformation of HKFl_2_ into KFl upon electrolyte contact is not unexpected considering the structural similarities between both phases. In particular, the stacking arrangement of the FlCO_2_^–^ anions is essentially similar in HKFl_2_ and KFl, suggesting that the proton–potassium exchange can occur without major rearrangement of the organic anionic framework. Such structural similarity makes a topotactic transformation in the solid state plausible, involving mainly local reorganization of the hydrogen-bonded carboxyl groups and incorporation of potassium ions.

In addition, the relatively small Gibbs free energy associated with Reaction R1 suggests that both phases are thermodynamically close in stability. This small energy difference may facilitate the H^+^/K^+^ exchange process in the presence of a potassium-rich electrolyte, promoting the conversion of HKFl_2_ into the fully potassiated KFl phase. This behavior also explains why KFl is the electrochemically active phase observed during cycling in all electrodes.

Figure 6b shows the evolution of the ex situ PXRD pattern of KFl during the first electrochemical cycle (Figure 6c). After the initial reduction, a pronounced amorphization of the structure is observed. Only the first reflection, assigned to the (110) family of lattice planes (Figure S12), remains visible, and it is slightly shifted to lower angles. This shift indicates an increase in the associated *d*_110_ spacing, consistent with structural expansion during K^+^ uptake and substantial rearrangement of the KFl framework upon reduction. Together with the lack of obvious vacant crystallographic sites in the pristine structure, this pronounced amorphization suggests that K^+^ uptake is not simply associated with the occupation of pre-existing sites. Instead, it is likely coupled to reduction-induced structural rearrangement, which may generate additional local coordination environments for ion accommodation.

Upon subsequent oxidation, the (110) reflection recovers its original position and several of the most intense peaks re-emerge (Figure 6b). This behavior indicates that the expansion associated with the (110) spacing is largely reversible during the first cycle. However, the pristine PXRD pattern is not fully restored after charge: the recovered reflections are less intense than in pristine KFl, indicating that the original long-range order is only incompletely recovered. Therefore, although substantial structural recovery occurs upon oxidation, and the loss of crystallinity could be due to material dissolution, the presence of residual K^+^ located in locally disordered oxygen-rich environments and/or irreversible rearrangement of the molecular packing cannot be fully excluded. More generally, the pronounced loss of diffraction intensity after reduction indicates that the long-range crystalline organization of pristine KFl is strongly disrupted during potassiation. As a result, the regular π–π stacked arrangement observed in the crystal structure cannot be considered to remain as a long-range ordered motif after reduction and is only partially recovered upon charge. Taken together, all these results indicate that KFl is the electrochemically active phase in all three cases, and the galvanostatic curves discussed above correspond to its electrochemical response.

These results also raise the question of whether analogous acid salt phases could form with other cations (e.g., Na^+^), or in positional isomers of the fluorenone core, which could influence the electrochemical behavior. In this context, previous studies on related quinone-based systems^39^ have shown that positional isomerism (e.g., ortho vs para substitution) can result in significant redox potential differences, highlighting the importance of molecular arrangement in determining electrochemical properties. From an application perspective, and considering the capacity retention observed in this work, increasing the number of carboxylate functionalities may enhance the ionic character and polarity of the compound, which is expected to reduce its solubility in the electrolyte and improve cycling stability.^40^

### Complementary Cytotoxicity Assessment

Preliminary cytotoxicity was assessed by 3-(4,5-dimethylthiazol-2-yl)-2,5-diphenyltetrazolium bromide (MTT) assay using human dermal fibroblasts (HDFn) (Figure S13). For all fluoren-9-one derived compounds, relative cell viability remained above approximately 75% across the tested concentration range (1–100 μM). According to ISO 10993-5 standard (ISO 10993-5:2009, Biological evaluation of medical devices—Part 5: tests for in vitro cytotoxicity), a reduction in cell viability greater than 30% relative to the untreated control (i.e., viability <70%) is generally considered indicative of cytotoxicity. Under our experimental conditions, none of the investigated compounds crossed this threshold, indicating the absence of marked cytotoxic effects in this assay.

## Conclusions

In this work, potassium derivatives of 9-oxo-9*H*-fluoren-4-carboxylic acid were synthesized, structurally characterized and evaluated in potassium half-cells. Two distinct crystalline phases can be selectively obtained from the parent acid by controlling the reaction conditions, particularly the HFl/KOH ratio and the solvent: the fully deprotonated potassium salt (KFl) and a hydrogen potassium carboxylate phase (HKFl_2_), together with mixtures of both. While acid salts of monoprotic carboxylic acids are well-known, the present results demonstrate that this fluorenone-based system allows selective access to two distinct potassium-containing crystalline phases depending on the reaction conditions.

The crystal structures of both salts are based on extended K–O coordination networks, whereas HKFl_2_ additionally exhibits strong O–H···O^–^ hydrogen bonds linking neutral and deprotonated molecules. The coexistence of these species in HKFl_2_, undetectable by solution NMR, is further supported by solid-state NMR spectroscopy.

Electrochemical measurements demonstrate that KFl is the electrochemically active phase in potassium half-cells, whereas HKFl_2_ converts into KFl upon contact with the electrolyte, most likely through a proton–potassium exchange process that can occur with minimal structural rearrangement due to the similarity of the stacking motifs in both structures. Under galvanostatic cycling in the 0.5–2.5 V vs K^+^/K window, KFl delivers a discharge capacity of ∼150 mAh/g in the second cycle, although the relatively low Coulombic efficiency (67%) indicates the presence of significant irreversible processes. These are likely related to a combination of active-material dissolution, reactions of soluble species, electrolyte/carbon/K-metal side processes and structural rearrangements. Among the electrolytes investigated, the best electrochemical performance is obtained using 2.5 M KFSI in TEP.

Overall, these results show that subtle differences in protonation state, governed by synthetic conditions, lead to distinct crystalline phases and directly influence the electrochemical behavior. More broadly, this work highlights how protonation equilibria, solid-state phase composition and electrolyte compatibility can critically affect the performance of simple aromatic carboxylates as organic electrode materials, providing insight for the rational design of more stable and efficient systems.

## Experimental Section

### Synthesis

**KFl**: 9-Oxo-9*H*-fluoren-4-carboxylic acid (1.77 mmol, 1.00 equiv mol) was added to an aqueous solution of KOH (1.52 mmol, 0.85 equiv) in H_2_O. The resulting suspension was stirred at ambient temperature for 8 h. The undissolved excess acid was removed by vacuum filtration, and the filtrate was concentrated under reduced pressure to afford the fully deprotonated potassium salt (90% yield). ^**1**^ **H NMR** (DMSO-*d*_6_, 400 MHz): δ 8.47 (dt, *J* = 7.6, 0.9 Hz, 1H), 7.59 (dd, *J* = 7.6, 1.3 Hz, 1H), 7.54 (dt, *J* = 7.3, 1.0 Hz, 1H), 7.50 (td, *J* = 7.6, 1.3 Hz, 1H), 7.43 (dd, *J* = 7.2, 1.3 Hz, 1H), 7.29 (td, *J* = 7.4, 1.0 Hz, 1H), 7.22 (t, *J* = 7.4 Hz, 1H); ^**13**^ **C NMR** (DMSO-*d*_6_, 101 MHz): δ 193.67, 169.82, 144.85, 140.65, 138.57, 135.08, 134.70, 133.52, 133.40, 128.38, 128.20, 125.85, 122.90, 121.78; **IR** ν (cm^–1^): 1717, 1607, 1571, 1466, 1450, 1419, 1397, 1370, 1299, 1251, 1243, 1193, 1165, 1086, 1072, 957, 870, 826, 803, 774, 763, 727, 690, 670, 630, 593, 564, 513, 438, 431; **MS (ESI** ^**–**^ **)** *m*/*z*: 179.0503 ([Fl]^−^, 89.2%), 223.0401 ([FlCO_2_]^−^, 100%), 469.0689 ([2FlCO_2_ + 2Na]^−^, 57.4%), 715.0974 ([3FlCO_2_ + 2Na]^−^, 20.4%).

**HKFl**_**2**_: 9-Oxo-9*H*-fluoren-4-carboxylic acid (0.65 mmol, 1 equiv) was added to a solution of KOH (0.29 mmol, 0.45 equiv mol) in THF/H_2_O (3:1, v/v). The solution was stirred at ambient temperature for 13 h, and the solvents were removed under reduced pressure. The residue was suspended in hot AcOEt and filtered hot under vacuum to give HKFl_2_ as a solid (96% yield). ^**1**^ **H NMR** (DMSO-*d*_6_, 400 MHz): δ 8.34 (dt, *J* = 7.7, 0.9 Hz, 1H), 7.73 (dd, *J* = 7.7, 1.3 Hz, 1H), 7.62–7.58 (m, 1H), 7.58 (dd, *J* = 7.3, 1.3 Hz, 1H), 7.55 (td, *J* = 7.6, 1.4 Hz, 1H), 7.36 (td, *J* = 7.4, 1.0 Hz, 1H), 7.33 (t, *J* = 7.5 Hz, 1H); ^**13**^ **C NMR** (DMSO-*d*_6_, 101 MHz): δ 192.98, 169.50, 143.77, 140.09, 135.31, 135.03, 133.85, 133.55, 129.12, 128.70, 125.69, 123.89, 123.32; **IR** ν (cm^–1^): 1719, 1713, 1608, 1586.5, 1569, 1471, 1450, 1444.5, 1419, 1367, 1359.5, 1296, 1279, 1242, 1191.5, 1165.5, 1154, 1095, 1090, 1072, 1049, 1029, 968.5, 958, 949.5, 920, 854, 875.5, 834.5, 807.5, 800, 782, 774, 770.5, 742.5 733, 722.5, 690, 670, 630, 588.5, 550.5, 564, 510, 443, 432.5, 404.5; **MS (ESI** ^**–**^ **)** *m*/*z*: 179.0503 ([Fl]^−^, 79,7%), 223.0401 ([FlCO_2_]^−^, 100%), 469.0689 ([2FlCO_2_ + 2Na]^−^, 67.5%), 715.0977 ([3FlCO_2_ + 2Na]^−^, 25.3%).

**HKFl**_**2**_ **+ KFl**: 9-Oxo-9*H*-fluoren-4-carboxylic acid (0.70 mmol, 1 equiv) was added to a solution of KOH (0.49 mmol, 0.70 equiv) in THF/H_2_O (3:1, v/v). After stirring the solution at ambient temperature for 7 h, the solvents were removed under reduced pressure. The residue was suspended in hot EtOH and collected by vacuum filtration to afford the phase mixture as a solid. ^**1**^ **H NMR** (DMSO-*d*_6_, 400 MHz): δ 8.38 (dt, *J* = 7.7, 0.9 Hz, 1H), 7.64 (dd, *J* = 7.7, 1.3 Hz, 1H), 7.57 (dt, *J* = 7.3, 1.1 Hz, 1H), 7.52 (td, *J* = 7.6, 1.3 Hz, 1H), 7.50 (dd, *J* = 7.2, 1.2 Hz, 1H), 7.33 (td, *J* = 7.4, 1.0 Hz, 1H), 7.27 (t, *J* = 7.5 Hz, 1H); ^**13**^ **C NMR** (DMSO-*d*_6_, 101 MHz): δ 193.35, 169.80, 144.34, 139.25, 138.05, 135.16, 134.84, 133.60, 133.53, 128.72, 128.42, 125.76, 123.09, 122.75; **IR** ν (cm^–1^): 1717, 1714.5, 1607, 1579.5, 1572, 1569, 1521, 1466, 1451, 1419, 1397, 1394, 1369.5, 1299, 1243, 1207, 1192.5, 1165, 1155, 1086, 1072.5, 957, 870, 827, 803.5, 774, 762.5, 725, 689, 670, 630.5, 592, 563.5, 552, 515, 438.5, 431.5; **MS (ESI** ^**–**^ **)** *m*/*z*: 179.0503 ([Fl]^−^, 95.3%), 223.0401 ([FlCO_2_]^−^, 100%), 469.0689 (2­[FlCO_2_ + Na]^−^, 57.4%, 67.5%), 715.0977 ([3FlCO_2_ + 2Na]^−^, 20.1%).

### Structural, Thermal and Morphological Characterization

Liquid ^**1**^ **H NMR** and ^**13**^ **C NMR** spectra were measured in a Bruker-400 spectrometer at room temperature; chemical shifts are reported in parts per million (δ) and coupling constants (*J*) are reported in hertz. ^1^H NMR spectra were referenced with respect to the residual proton signal of the deuterated solvent. ^13^C NMR spectra, proton uncoupled, were referenced to the chemical shifts of the deuterated solvent. Solid-state NMR measurements were carried out on a Bruker Avance NEO 400 WB spectrometer (9.4 T) using a 4 mm MAS probe. ^1^H MAS and ^13^C CP/MAS spectra were recorded at a spinning frequency of 12 kHz. SPINAL-64 decoupling was applied during ^13^C acquisition. ^13^C chemical shifts were referenced to the carbonyl signal of external glycine (δ = 176.03 ppm), and ^1^H chemical shifts were referenced to the water signal (δ = 4.7 ppm) measured as an external reference. All measurements were performed at room temperature. Infrared (**IR**) spectra were measured in a PerkinElmer UATR Two spectrophotometer (PerkinElmer, Shelton, CT, USA), with a 2 cm^–1^ spectral resolution and a total of 8 scans. **MS** data were acquired on an Agilent 6200 Series TOF/6500 Series Q-TOF mass spectrometer equipped with an ESI source, using FIA mode in negative ionization. Data were processed with Agilent MassHunter Qualitative Analysis software. Powder X-ray diffraction (**PXRD**) data were collected on a PANalytical X’Pert PRO MPD diffractometer with Cu K_α_ radiation (λ_1_ = 1.540598 Å, λ_2_ = 1.544426 Å). The patterns were registered in the range 2° ≤ 2θ ≤ 60°, with a step size of 0.033°. Ex situ PXRD measurements were performed on a multipurpose Malvern PANalytical Empyrean Alpha1 diffractometer using monochromatic Cu K_α_ radiation (λ = 1.540598 Å) over the same 2θ range (2–60°), with a step size of 0.033°. Thermogravimetric analysis (**TGA**) was conducted using TA Q500 equipment (TA Instruments); samples were heated at 10 °C/min from room temperature to 800 °C under nitrogen atmosphere. Scanning electron microscopy (**SEM**) was performed using a JEOL 6400 JSM (JEOL Ltd., Tokyo, Japan) or a JEOL JSM IT700HR microscope (JEOL Ltd.); samples were coated with a thin film of gold prior to imaging.

### Crystallography

Single crystal X-ray diffraction data collection for compounds KFl and HKFl_2_ was carried out at room temperature on a Bruker D8 VENTURE system equipped with an ImuS3.0 Incoatec microfocus sealed tube (Cu K_α_, λ = 1.54178 Å) and a Montel multilayer optics monochromator.

Using Olex,^41^ the structures were solved with the SHELXS-1997 ^42^ structure solution program using direct methods and refined with the SHELXL^43^ refinement package using least squares minimization. All nonhydrogen atoms were refined anisotropically. All hydrogen atoms were included in their calculated positions and refined on their respective carbon or oxygen atoms.

A summary of the fundamental crystal and refinement data is given in Tables S2–S14 of the Supporting Information.

CCDC 2345911 and 2380849 deposition numbers for compounds (HKFl_2_) and (KFl) contain the crystallographic data for this paper. These data can be obtained free of charge from the Cambridge Crystallographic Data Centre via www.ccdc.cam.ac.uk/data_request/cif.

### Electrochemical Performance

Electrodes were prepared by mixing the active material with 25 wt % Super C-65 carbon (Imerys) and softly grinding the mixture with a mortar and pestle. The electrodes were vacuum-dried at 80 °C and subsequently transferred into an argon-filled glovebox (<1 ppm of H_2_O, <1 ppm of O_2_) for cell assembly. Swagelok-type half-cells were assembled by sequentially stacking 2–4 mg of the cathode mixture, a 13 mm glass fiber separator soaked with liquid electrolyte, and an 11 mm K metal disk on top of a stainless-steel plunger. Potassium disk electrodes were prepared by cutting a slice of a K cube (Sigma-Aldrich), removing the oxidized surface, rinsing off the oil with hexane, flattening the metal inside a polypropylene bag using a roller and cutting a disk with a puncher. The following five electrolytes were prepared by dissolving the corresponding potassium salt in the solvent or solvent mixture. In all cases, the molarity was calculated with respect to the volume of solvent used: (i) 2.5 M KFSI in triethyl phosphate (TEP); (ii) 3.9 M KFSI in dimethoxyethane (DME); (iii) 1 M KFSI in 1:1 (wt) ethylene carbonate/diethyl carbonate (EC/DEC); (iv) 1 M KFSI in 2-methyltetrahydrofuran (Me-THF); (v) 1 M KPF_6_ in triethyl phosphate (TEP). Cells were galvanostatically cycled at C/10 (10.2 mA/g for KFl and 11.0 for HKFl_2_ and the phase mixture).

Solubility tests in the electrolytes were performed by adding 1 mg of KFl to 0.5 mL of the corresponding electrolyte and leaving the mixture in contact for 48 h inside the glovebox. The solution was then separated from the remaining solid to improve the clarity of the photograph.

A blank electrode composed exclusively of Super C65 carbon powder was prepared as a qualitative reference. The C65 electrode had a total mass of 2.5 mg and was tested in a potassium half-cell using the same cell configuration, electrolyte, voltage window and cycling protocol as the KFl electrodes.

Ex situ XRD measurements were performed on electrodes recovered after the first discharge (0.5 V vs K^+^/K) and the first charge (2.5 V vs K^+^/K) in Swagelok half-cells. For sample preparation, the cells were disassembled inside the glovebox and the electrodes were carefully retrieved. The electrodes were washed three times with the corresponding electrolyte solvent to remove residual salts, then vacuum-dried. The resulting powders were gently ground, loaded into 0.5 mm glass capillaries, and flame-sealed prior to measurement.

### Computational Details

DFT calculations under static conditions have been performed using the Vienna ab initio simulation package (VASP)^44^ and the projector augmented wave (PAW method).^45^ We use the Perdew–Burke exchange correlation functional^46^ and the Grimme-D3 dispersion correction with Becke–Johnson damping function.^47^ A Plane wave basis set expansion with a kinetic energy cutoff of 900 eV with a Γ centered *k*-point mesh with a resolution of 0.20 Å^–1^ were used. Convergence criteria for the geometry optimizations were 1 × 10^–8^ eV in the electronic self-consistent loop and 5 × 10^–3^ eV/Å for the forces acting on the nuclei. Initial crystal structures were taken from the experimental X-ray data obtained in this work. Optimized structures are provided as cif files in the electronic Supporting Information. Their corresponding energies are reported in Table 3.

**Table 3:** Computed Energies per Unit Cell for HFl, HKFl_2_, and KFl

compound	unit cell stoichiometry	energy (eV/unit cell)
HFl	C_56_O_12_H_32_	–712.845
HKFl_2_	K_2_C_56_O_12_H_30_	–712.254
KFl	K_8_C_112_O_24_H_56_	–1423.134

### Toxicity

Human dermal fibroblasts (HDFn, PCS-201-010, ATCC) were cultured according to the supplier’s instructions. Cells were seeded in 96-well plates at a density of 2 × 10^4^ cells per well in 100 μL of culture medium supplemented with 10% fetal bovine serum (FBS, Gibco) and 1% penicillin–streptomycin (Sigma-Aldrich), and incubated overnight at 37 °C in a humidified atmosphere containing 5% CO_2_.

On the following day, the compounds were added and the cells were incubated for 48 h. After treatment, 10 μL of MTT solution were added to each well and incubated for an additional 2 h at 37 °C. The absorbance was measured at 570 nm using a microplate reader (BMG LABTECH). Cell viability was expressed as a percentage relative to untreated control cells. All experiments were performed in triplicate.

## Supplementary Material





## Notes

^13^C NMR spectra; additional ^1^H NMR spectra; thermogravimetric analysis data (including a table of main parameters); SEM micrographs; crystallographic details and refinement tables from single-crystal X-ray diffraction; illustration of C–H···π interactions; comparison between experimental and simulated PXRD patterns based on SCXRD data; electrochemical performance of KFl in different electrolytes; photographs showing the visual solubility/color changes of KFl in different electrolytes; electrochemical performance of the C65 blank electrode; view of the (110) plane in KFl; and cytotoxicity plot (PDF)
Calculated CIF files for DFT-optimized structures of HFl (FlOH_calc.cif), HKFl_2_ (Fl2O2HK_calc.cif), and KFl (FlOK_calc.cif) (ZIP)

## References

[1] Komaba S., Hasegawa T., Dahbi M., Kubota K. (2015). Potassium
Intercalation
into Graphite to Realize High-Voltage/High-Power Potassium-Ion Batteries
and Potassium-Ion Capacitors. Electrochem. Commun..

[2] Li M., Wang C., Wang C., Lyu Y., Wang J., Xia S., Mao J., Guo Z. (2025). 10 Years Development
of Potassium-Ion
Batteries. Adv. Mater..

[3] Wang L., Zhang S., Li N., Chen J., Chen Y., Zhang Z., Tan L., Niu X., Yang Y., Zhang J., Li H., Ji X., Zhu Y. (2024). Prospects
and Challenges of Practical Nonaqueous Potassium-Ion Batteries. Adv. Funct. Mater..

[4] Wei W., Zhang C., Yuan X., Zhang J. (2025). Organic Electrode Materials
for Lithium/Sodium/Potassium-Ion Batteries: Synthesis, Characterizations,
Functional Mechanisms, and Performance Validation. Electrochem. Energy Rev..

[5] Zarrabeitia M., Carretero-González J., Leskes M., Adenusi H., Iliev B., Schubert T. J. S., Passerini S., Castillo-Martínez E. (2023). Could Potassium-Ion
Batteries Become
a Competitive Technology?. Energy Mater..

[6] Hu Y., Fan L., Lu B., Yin S. F. (2025). Designing Better
Organic Electrodes
for Potassium Ion Batteries. ChemSusChem.

[7] Hari
Prasad P. M., Malavika G., Pillai A., Sadan S., Pillai Z. S. (2024). Emerging Organic Electrode Materials for Sustainable
Batteries. NPG Asia Mater..

[8] Khobrekar P., Bugde S. (2025). Recent Advances of
9-Fluorenone in Photoredox Catalysis. Mol. Catal..

[9] Junaid H. M., Waseem M. T., Khan Z. A., Munir F., Sohail S., Farooq U., Shahzad S. A. (2022). Fluorenone-Based
Fluorescent and
Colorimetric Sensors for Selective Detection of I^–^ Ions: Applications in HeLa Cell Imaging and Logic Gate. ACS Omega.

[10] Svirskaite L. M., Kasparavičius E., Steponaitis M., Grzibovskis R., Franckevičius M., Katerski A., Naujokaitis A., Karazhanov S., Gopi S. V., Aizstrauts A., Vembris A., Getautis V., Malinauskas T. (2024). Fluorene-
and Fluorenone-Based Molecules as Electron-Transporting SAMs for Photovoltaic
Devices. RSC Adv..

[11] Daniel
Thangadurai T., Nithya I., Manjubaashini N., Bhuvanesh N., Bharathi G., Nandhakumar R., Nataraj D. (2018). Fluorenone-Based Fluorescent Probe for Selective “Turn-On”
Detection of Pyrophosphate and Alanine. Spectrochim.
Acta, Part A.

[12] Cihaner A., Tirkeş S., Önal A. M. (2004). Electrochemical Polymerization of
9-Fluorenone. J. Electroanal. Chem..

[13] Neha, Kaur N. (2024). Unveiling the Versatile
Applications of 9-Fluorenone: A Comprehensive Review 2010–2024. Coord. Chem. Rev..

[14] Semin S., Li X., Duan Y., Rasing T. (2021). Nonlinear Optical Properties and
Applications of Fluorenone Molecular Materials. Adv. Opt. Mater..

[15] Wei X., Pan W., Duan W., Hollas A., Yang Z., Li B., Nie Z., Liu J., Reed D., Wang W., Sprenkle V. (2017). Materials
and Systems for Organic Redox Flow Batteries: Status and Challenges. ACS Energy Lett..

[16] Feng R., Zheng X., Rice P. S., Bazak J. D., Hollas A., Shao Y., Liang Y., Wang W. (2024). Redox Activity Modulation
in Extended Fluorenone-Based Flow Battery Electrolytes with π-π
Stacking Effect. J. Electrochem. Soc..

[17] Rodriguez J., Niemet C., Pozzo L. (2019). Fluorenone Based Anolyte
for an Aqueous
Organic Redox-Flow Battery. ECS Trans..

[18] Tobishima S., Yamaki J., Yamaji A. (1984). Cathode Characteristics
of Organic
Electron Acceptors for Lithium Batteries. J.
Electrochem. Soc..

[19] Lv L., Hu Z., An N., Xie K., Yang Y., Zhang Z., Li Z. (2021). A Green and Sustainable
Organic Molecule Electrode Prepared by Fluorenone
for More Efficient Energy Storage. Electrochim.
Acta.

[20] Soni C. B., Bera S., Sungjemmenla, Vineeth S. K., Kumar H., Kumar V. J. (2023). Novel Organic
Molecule Enabling a Highly Stable and Reversible Sodium Metal Anode
for Room-Temperature Sodium-Metal Batteries. J. Energy Storage.

[21] Clare
Speakman J. (1972). Acid Salts of Carboxylic Acids, Crystals with Some
“Very Short” Hydrogen Bonds. Struct.
Bonding.

[22] Jayasheela K., Nagabalasubramanian P. B., Periandy S. (2020). Conformational &
Spectroscopic Characterization, Charge Analysis and Molecular Docking
Profiles of Chromone-3-Carboxylic Acid Using a Quantum Hybrid Computational
Method. Heliyon.

[23] Shurvell, H. F. Spectra–Structure Correlations in the Mid- and Far-Infrared. In Handbook of Vibrational Spectroscopy; Wiley, 2006.

[24] Oomens J., Steill J. D., Redlich B. (2009). Gas-Phase
IR Spectroscopy of Deprotonated
Amino Acids. J. Am. Chem. Soc..

[25] Socrates, G. Infrared and Raman Characteristic Group Frequencies: Tables and Charts, 3rd ed.; Wiley: Chichester, 2004.

[26] Nakamoto, K. ; McCarthy, P. J. Spectroscopy and Structure of Metal Chelate Compounds; Wiley: New York, 1968.

[27] Claridge, T. D. W. High-Resolution NMR Techniques in Organic Chemistry; Elsevier: Oxford, 2016; pp 11–59.

[28] Kennard C. H. L., Katekar G. F. (1981). Crystal Structure of 9-Oxofluorene-4-carboxylic Acid. Aust. J. Chem..

[29] Macrae C.
F., Bruno I. J., Chisholm J. A., Edgington P. R., McCabe P., Pidcock E., Rodriguez-Monge L., Taylor R., van de Streek J., Wood P. A. (2008). Mercury CSD 2.0
- New Features for the Visualization and Investigation of Crystal
Structures. J. Appl. Crystallogr..

[30] Bernstein J., Davis R. E., Shimoni L., Chang N. L. (1995). Patterns
in Hydrogen
Bonding: Functionality and Graph Set Analysis in Crystals. Angew. Chem., Int. Ed. Engl..

[31] Luss H. R., Smith D. L. (1972). The Crystal and Molecular Structure of 9-Fluorenone. Acta Crystallogr., Sect. B.

[32] Momma K., Izumi F. (2011). VESTA 3 for Three-Dimensional Visualization
of Crystal, Volumetric
and Morphology Data. J. Appl. Crystallogr..

[33] Duer, M. J. Solid-State NMR Spectroscopy: Principles and Applications; Wiley-Blackwell: Oxford, 2004.

[34] Harris R. K. (2007). Applications
of Solid-State NMR to Pharmaceutical Polymorphism and Related Matters. J. Pharm. Pharmacol..

[35] Castillo-Martínez E., Solana-Madruga E., Ebrahimi-Koodehi S., Leskes M., del Burgo-Olivares C., Linage M., Martín C., Sanchez-Ahijón E., Gómez-Herrero A., Cascos V. (2024). Hybrid Carboxylate-Schiff
Bases as Electroactive Anode Materials for Potassium-Ion Batteries. Sustainable Mater. Technol..

[36] Zhou M., Bai P., Ji X., Yang J., Wang C., Xu Y. (2021). Electrolytes
and Interphases in Potassium Ion Batteries. Adv. Mater..

[37] Xu Y., Ding T., Sun D., Ji X., Zhou X. (2023). Recent Advances
in Electrolytes for Potassium-Ion Batteries. Adv. Funct. Mater..

[38] Hosaka T., Muratsubaki S., Kubota K., Onuma H., Komaba S. (2019). Potassium
Metal as Reliable Reference Electrodes of Nonaqueous Potassium Cells. J. Phys. Chem. Lett..

[39] Sieuw L., Lakraychi A. E., Rambabu D., Robeyns K., Jouhara A., Borodi G., Morari C., Poizot P., Vlad A. (2020). Through-Space
Charge Modulation Overriding Substituent Effect: Rise of the Redox
Potential at 3.35 V in a Lithium-Phenolate Stereoelectronic Isomer. Chem. Mater..

[40] Zhao Q., Guo C., Lu Y., Liu L., Liang J., Chen J. (2016). Rechargeable
Lithium Batteries with Electrodes of Small Organic Carbonyl Salts
and Advanced Electrolytes. Ind. Eng. Chem. Res..

[41] Dolomanov O. V., Bourhis L. J., Gildea R. J., Howard J. A. K., Puschmann H. (2009). OLEX2: A Complete
Structure Solution, Refinement and Analysis Program. J. Appl. Crystallogr..

[42] Sheldrick G. M. A. (2008). Short
History of SHELX. Acta Crystallogr. Sect. A:
Fundam. Crystallogr..

[43] Sheldrick G. M. (2015). Crystal
Structure Refinement with SHELXL. Acta Crystallogr.,
Sect. C: Struct. Chem..

[44] Kresse G., Furthmüller J. (1996). Efficiency
of ab Initio Total Energy Calculations for
Metals and Semiconductors Using a Plane-Wave Basis Set. Comput. Mater. Sci..

[45] Blöchl P. E. (1994). Projector
Augmented-Wave Method. Phys. Rev. B: Condens.
Matter Mater. Phys..

[46] Perdew J. P., Burke K., Ernzerhof M. (1996). Generalized
Gradient Approximation
Made Simple. Phys. Rev. Lett..

[47] Grimme S., Antony J., Ehrlich S., Krieg H. (2010). A Consistent and Accurate
ab Initio Parametrization of Density Functional Dispersion Correction
(DFT-D) for the 94 Elements H–Pu. J.
Chem. Phys..

