# Supplementary material for: Potassium Salts of 9‑Oxo‑9H‑fluoren-4-carboxylic Acid: Phase Formation, Crystal Structure, and Electrochemical Behavior in Potassium-Ion Batteries
Source: Chem Mater. 2026 Sep 10;38(18):9288–99. doi: 10.1021/acs.chemmater.6c01146 (PMC13688338; doi:10.1021/acs.chemmater.6c01146)
Supplement: Supplementary file 1 [file cm6c01146_si_001.pdf]

Potassium Salts of 9-Oxo-9*H*-fluoren-4-carboxylic Acid: Phase Formation, Crystal Structure, and Electrochemical Behaviour in Potassium-Ion Batteries

Irene Gómez-Berenguer (https://orcid.org/0009-0007-5459-0439),[1,2] Patricia Delgado-Martínez (https://orcid.org/0000-0002-6984-8152),[3] Álvaro Lobato (https://orcid.org/0000-0002-2798-6178),[4], Bernardo Herradón (https://orcid.org/0000-0003-1179-884X),[2] Elizabeth Castillo-Martínez (https://orcid.org/0000-0002-8577-9572).[1,*]

[1] Departamento de Química Inorgánica, Universidad Complutense de Madrid, 28040, Madrid, Spain; ecastill@ucm.es (E.C.M); iregom12@ucm.es (I.G.B.)

[2] Instituto de Química Orgánica General, Consejo Superior de Investigaciones Científicas, 28006, Madrid, Spain; b.herradon@csic.es (B.H.); irene.gomez.berenguer@csic.es (I.G.B)

[3] Unidad de Difracción de Rayos X, Centro de Asistencia a la Investigación de Técnicas Químicas, Universidad Complutense de Madrid, 28040, Madrid, Spain; patriciadelgado@ucm.es (P.D.M)

[4] Departamento de Química Física, Universidad Complutense de Madrid, 28040, Madrid, Spain; a.lobato@ucm.es (A.L.)

* Correspondence: b.herradon@csic.es, ecastill@ucm.es

Contents

1. **Spectroscopic characterization** 2
2. **Thermal analysis** 4
3. **Morphological characterization** 5
4. **Crystal structure details** 5
5. **Electrochemical performance** 22
6. **Toxicity** 24

1. Spectroscopic characterization

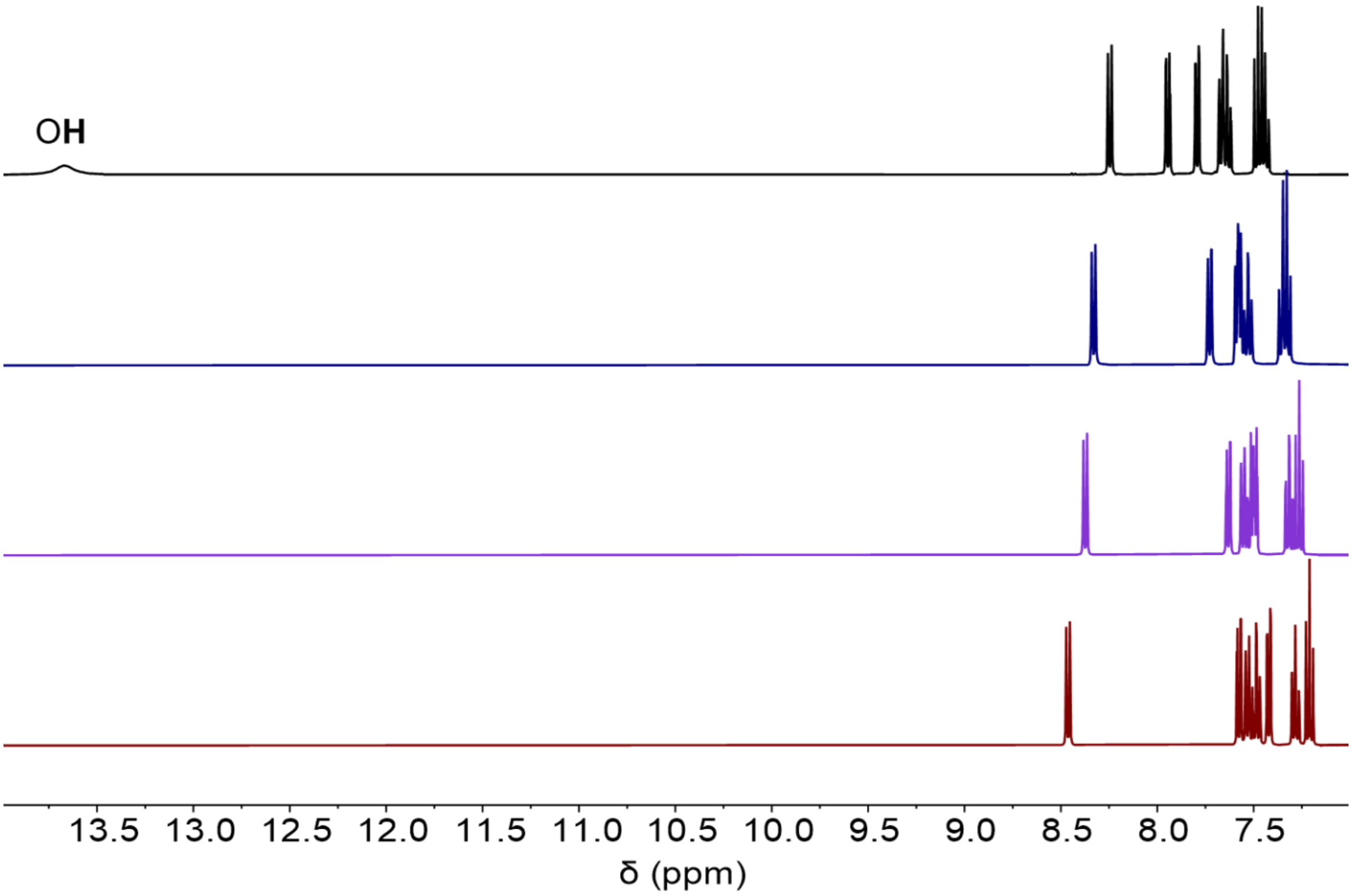

Fig. S1. ^{1}H-NMR (DMSO-d_6)

— HFI — $HKFI_2$ — KFI + $HKFI_2$ — KFI

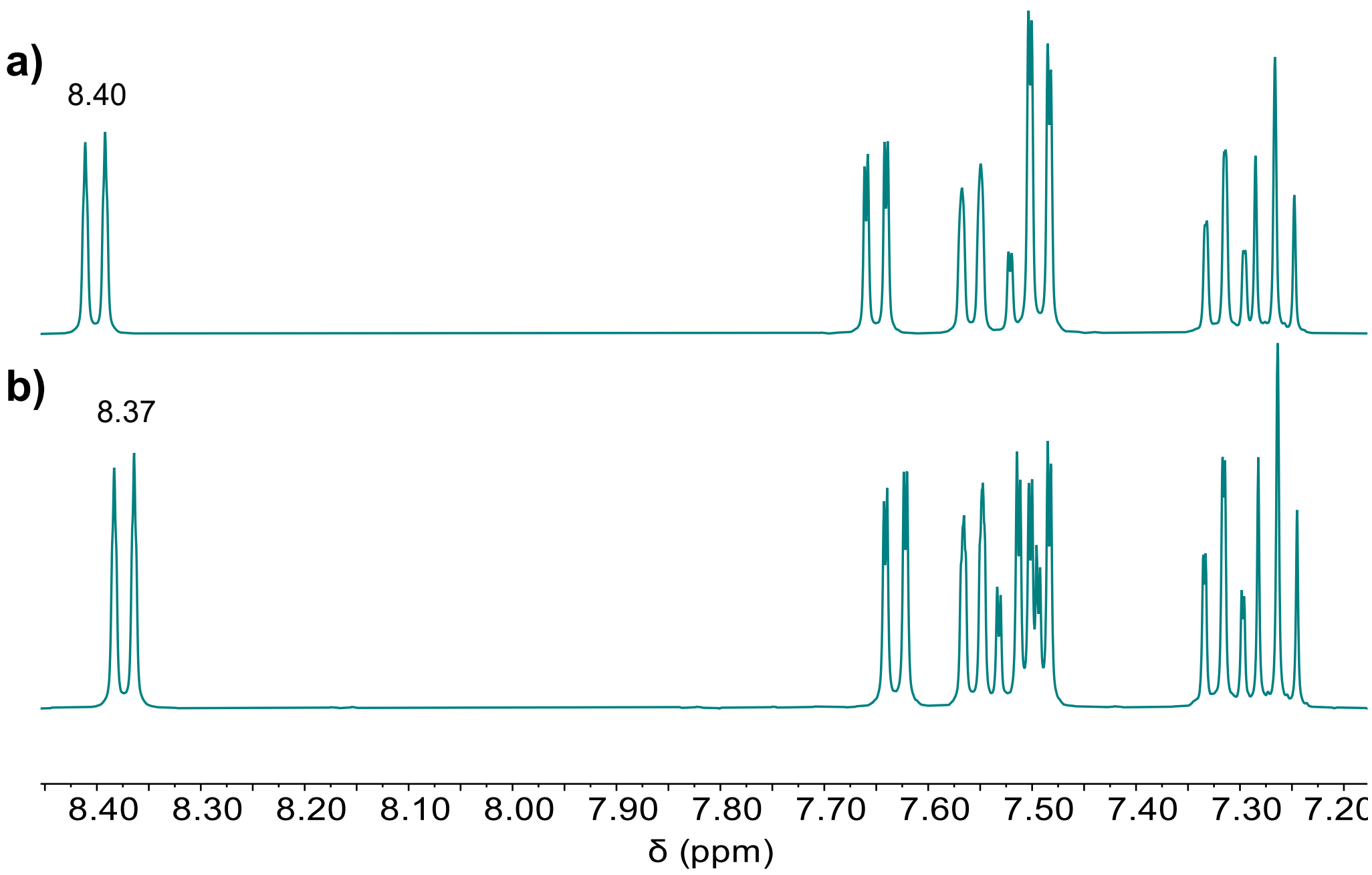

Fig. S2. Comparison of two independent ^{1}H-NMR measurements of the KFI + $HKFI_2$ phase mixture in DMSO-d_6 recorded at different times.

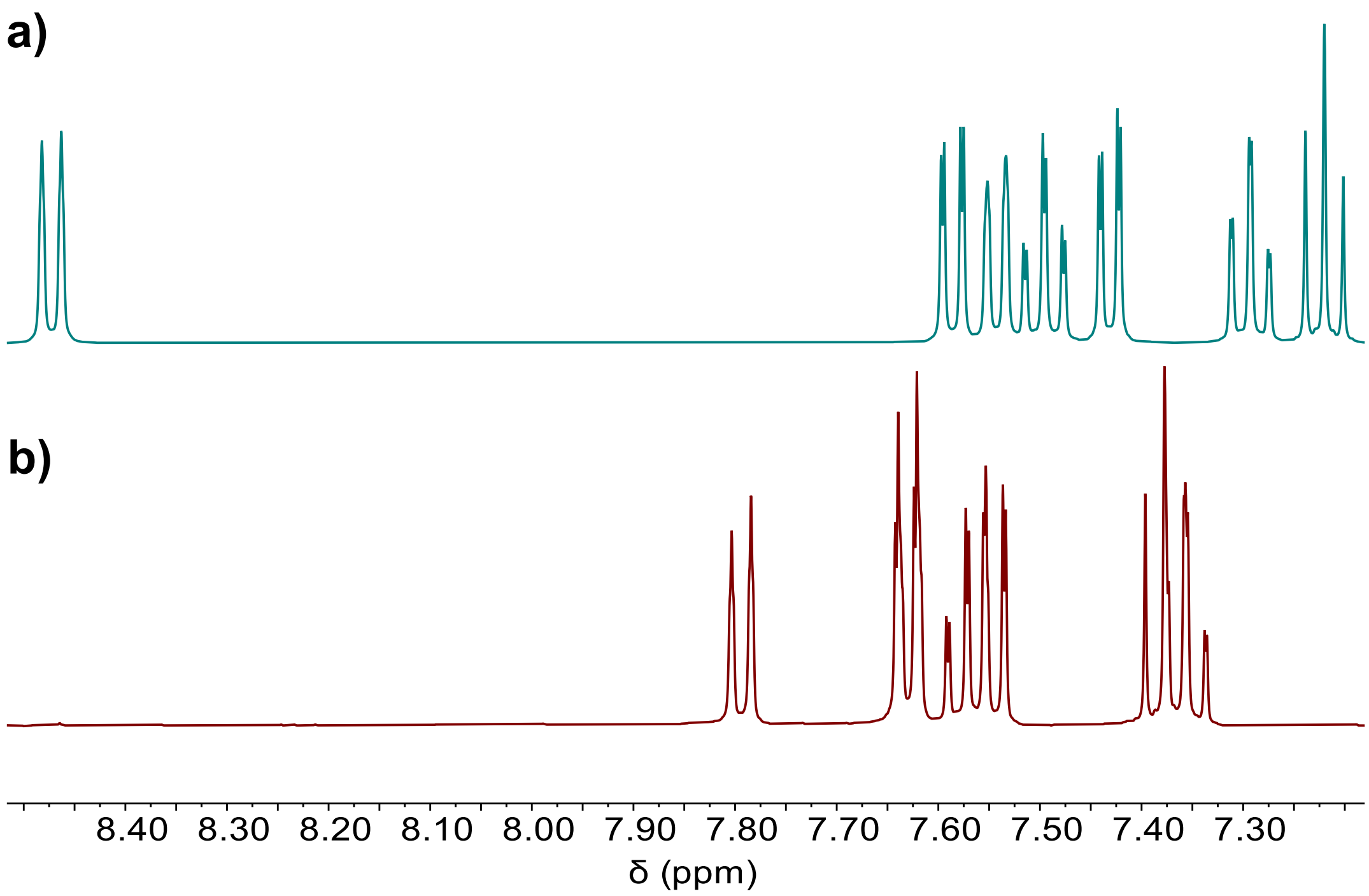

Fig. S3. ^{1}H-NMR KFI in (a) DMSO-*d*$_6$ and (b) D_2O.

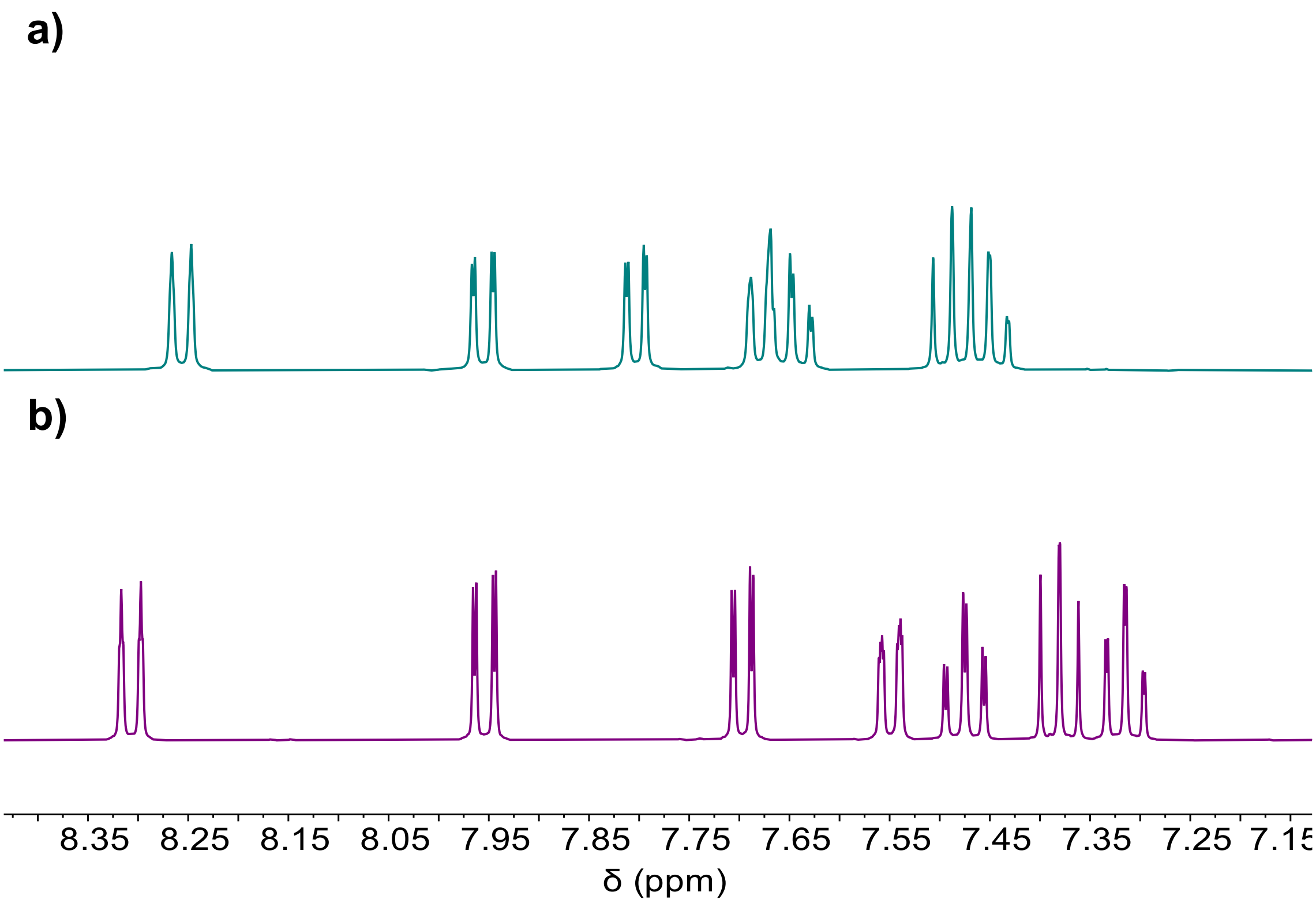

Fig. S4. ^{1}H-NMR HFI in (a) DMSO-*d*$_6$ and (b) acetone-d$_6$.

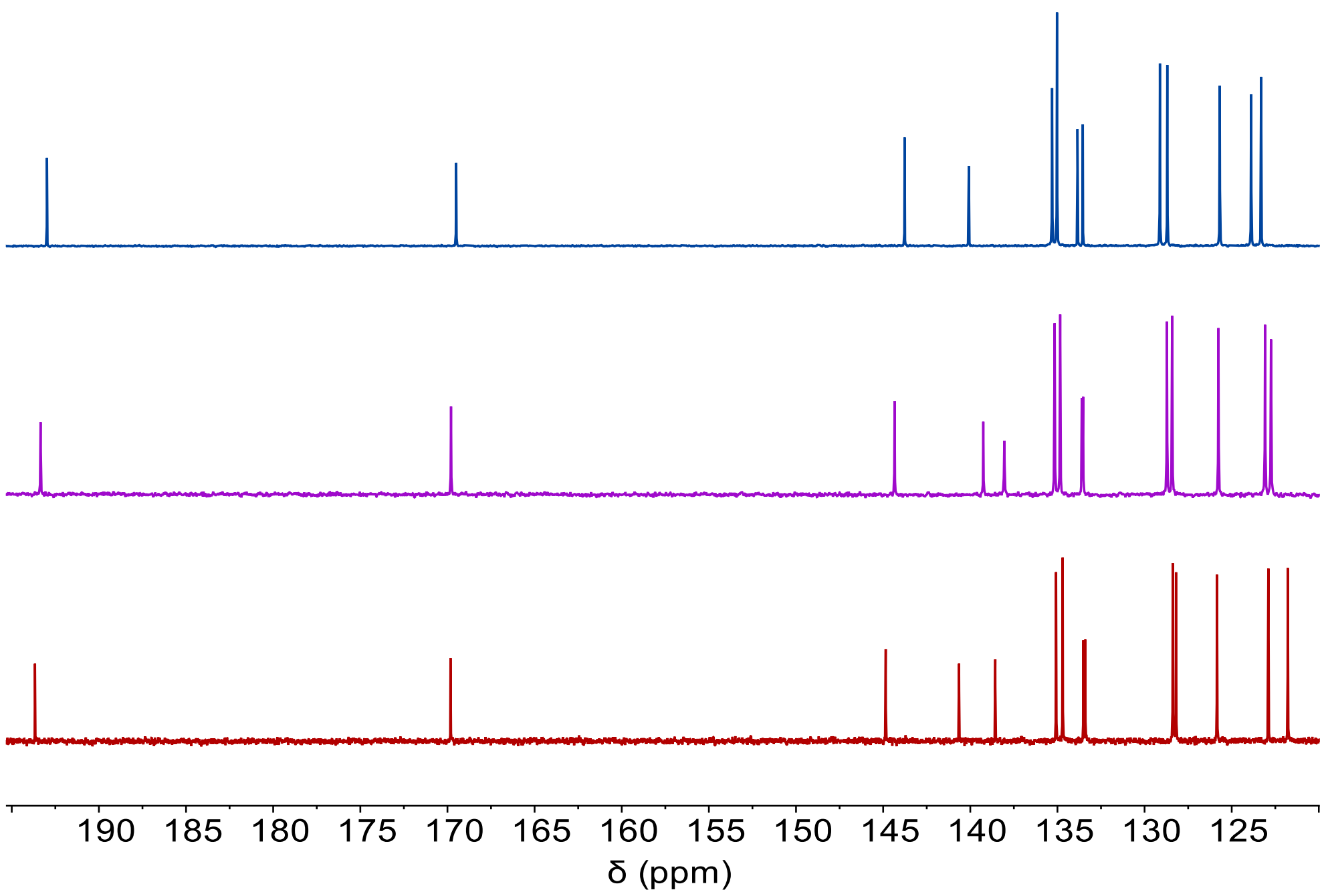

Fig. S5. ^{13}C-NMR (DMSO-d_6)

— $HKFI_2$ — KFI + $HKFI_2$ — KFI

2. Thermal analysis

Table S1. Thermogravimetric parameters.

	T_{onset} (°C)	T_p (°C)	Mass loss (%)	Residue (%)
HFI	~250	~298	~100	~0
KFI	~449	~462	~17	~82
$HKFI_2$ (step 1)	~284	~312	~41	—
$HKFI_2$ (step 2)	~454	~458	~10	~49
Mixture (step 1)	~263	~287	~15	—
Mixture (step 2)	~446	~461	~14	~71

In $HKFI_2$, the first mass-loss step (~41%) is close to the theoretical value for release of one HFI unit from a 1:1 HFI·KFI assembly (~46%), supporting its assignment to acid liberation.

3. Morphological characterization

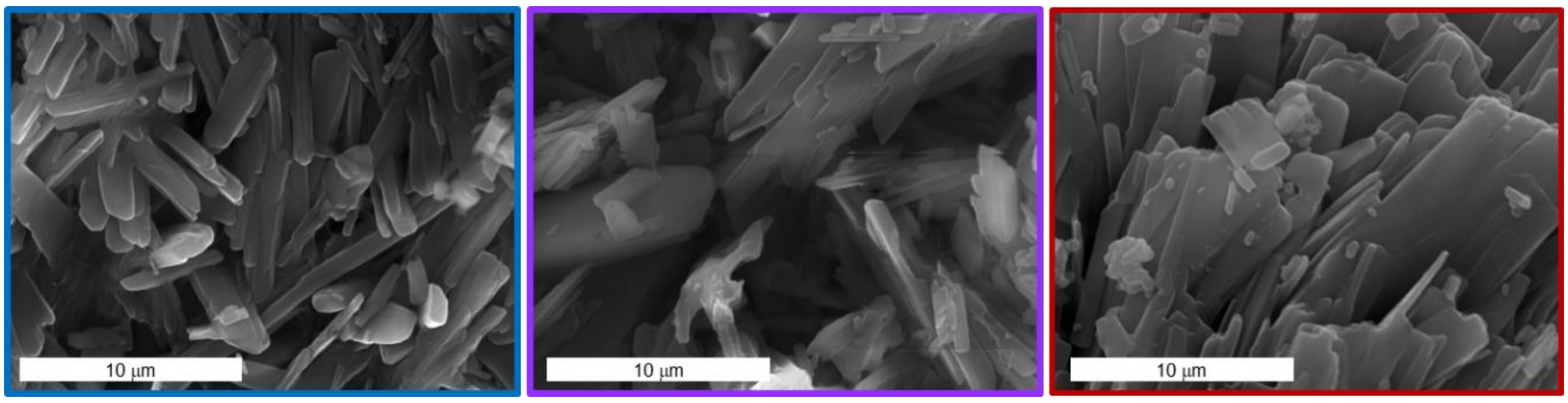

Fig. S6. SEM micrographs.

— $HKFI_2$ — KFI + $HKFI_2$ — KFI

4. Crystal structure details

Table S2. Crystallographic parameters of KFI and $HKFI_2$ [a,b].

	**KFI**	**$HKFI_2$**
Formula	$C_{28}H_{14}K_2O_6$	$C_{28}H_{15}KO_6$
M/g/mol	524.59	486.50
Crystal system	Monoclinic	Triclinic
Space group	*P*2$_1$/*c*	*P*-1
Temperature/K	*306.00*	*273.15*
a/Å	17.2425(4)	6.9723(2)
b/Å	18.9409(5)	11.8536(4)
c/Å	7.07880(10)	13.4853(5)
α/°	90	82.065(2)
β/°	94.143(2)	84.593(2)
γ/°	90	84.943(2)
Z	4	2
Z'	1	1
V/Å^3	2305.81	1095.74
P_{calc}/g/cm^{-3}	1.511	1.475
μ/mm^{-1}	4.016	2.510
F(000)	1072.0	500.0
Crystal size/mm^3	0.094x0.054x0.017	0.051x0.034x0.026
2Θ range for data collection/º	5.138 to 159.706	6.64 to 159.346
Reflections collected	154037	4680
Data/renstraint/parameters	4998/0/326	4680/0/320
Goodness-of.fit on F^2	1.011	1.066
Final R indexes [I>2σ (I)]	R_1=0.0634, wR_2=0.1640	R_1=0.0564, wR_2=0.1414
Final R indexes [all data]	R_1=0.0980, wR_2=0.1984	R_1=0.0827, wR_2=0.1554
Largest diff. peak/hole/e Å^{-3}	0.80/-0.42	0.28/-0.30
R-Factor (%)	6.8	5.64

[a] Common details: radiation Cu Kα (λ = 1.54178).
[b] CCDC deposition numbers: 2380849 for KFI and 2345911 for $HKFI_2$.

Note that all structural details listed in the tables below correspond to the experimentally determined crystal structures from single crystal X-ray diffraction. cif files for the DFT optimized crystal structures are provided separately, but details are not included in this document.

Table S3. Fractional Atomic Coordinates (×10^4) and Equivalent Isotropic Displacement Parameters (Å^2×10^3) for **KFI** (CCDC 2380849). U_{eq} is defined as 1/3 of the trace of the orthogonalised U_{IJ} tensor.

Atom	*x*	*y*	*z*	U(eq)
K1	3340.5(5)	3181.8(4)	8368.5(11)	69.3(3)
K2	-1678.2(5)	6849.6(4)	6458.6(10)	66.8(3)
O1	6871.6(18)	6723.1(15)	7832(5)	95.8(9)
O2	3713.0(16)	4561.9(15)	8251(4)	85.1(8)
O3	-1354.8(15)	5458.4(14)	6414(3)	77.9(7)
O4	1819.0(15)	3300.0(13)	7421(4)	74.7(7)
O5	1092.0(18)	3127.9(14)	9844(4)	87.0(8)
C1	6384(2)	6509(2)	6605(5)	69.1(9)
C2	6045.3(19)	5784.5(17)	6906(4)	61.4(8)
C3	5284.5(19)	5683.4(17)	7385(4)	59.4(7)
C4	4981.2(19)	4992.7(17)	7528(4)	60.4(7)
C5	4164(2)	5042.6(18)	7999(5)	66.1(8)
C6	1346(2)	3493.0(18)	8533(4)	62.5(8)
C7	1002.1(18)	4230.9(16)	8280(4)	57.3(7)
C8	250.5(18)	4336.5(16)	7521(4)	55.4(7)
C9	-69.0(19)	5021.5(17)	7455(4)	57.5(7)
C10	-889(2)	4979.1(18)	6662(4)	64.3(8)
C11	-1651(3)	3188(2)	4964(6)	87.3(12)
C12	-1681(2)	3898(2)	5355(5)	77.8(10)
C13	-1032.2(19)	4213.8(18)	6214(4)	63.4(8)
C14	350(2)	5601.5(18)	8128(4)	65.1(8)
C15	1112(2)	5491.6(18)	8834(5)	66.2(8)
C16	1429(2)	4819.1(19)	8898(5)	64.5(8)
O17	6143(3)	6843.1(16)	5167(5)	117.4(13)
C18	3996(2)	5809.5(19)	8170(5)	66.7(8)
C19	4671(2)	6187.7(18)	7830(4)	64.5(8)
C20	5417(2)	4407.4(19)	7192(5)	69.8(9)
C21	6186(2)	4503(2)	6764(5)	74.4(9)
C22	6488(2)	5186(2)	6631(5)	70.7(9)
C23	4671(3)	6916(2)	8042(6)	79.9(10)
C24	3990(3)	7233(3)	8544(6)	95.7(13)
C25	3329(3)	6863(3)	8821(7)	96.2(14)
C26	3329(2)	6138(3)	8646(6)	85.7(11)
C27	-346.3(19)	3840.9(18)	6699(4)	60.4(7)
C28	-319(2)	3124.0(19)	6291(5)	71.7(9)
C29	-978(3)	2808(2)	5429(6)	88.9(12)

Table S4. Fractional Atomic Coordinates (×10⁴) and Equivalent Isotropic Displacement Parameters (Å²×10³) for **HKFI₂** (CCDC 2345911). U_{eq} is defined as 1/3 of the trace of the orthogonalised U_{IJ} tensor.

Atom	*x*	*y*	*z*	U(eq)
K1	7550.0(8)	9075.7(5)	4945.9(5)	55.3(2)
O1A	2489(3)	3123.3(16)	5388.4(18)	71.9(6)
O1B	13140(3)	8335(2)	10414.7(18)	85.5(7)
O2A	1445(3)	8696.0(15)	4887.9(16)	59.7(5)
O2B	7614(4)	8833(2)	6926.9(17)	86.9(8)
O3A	3676(3)	8720.8(16)	5954.4(16)	62.9(5)
O3B	4971(3)	8088.6(19)	7680.5(18)	68.1(6)
C1A	1635(4)	4767(2)	6983(2)	59.3(7)
C1B	9632(5)	6988(3)	10285(2)	70.8(8)
C2A	1378(4)	5682(3)	7529(2)	61.5(7)
C2B	7982(5)	6539(3)	10072(3)	78.5(9)
C3A	1648(4)	6775(2)	7057(2)	56.4(7)
C3B	7008(5)	7056(3)	9254(2)	67.5(8)
C4A	2112(3)	7008(2)	6022(2)	46.7(6)
C4B	7660(4)	8008(2)	8626(2)	55.0(6)
C5A	2290(3)	6096(2)	5461(2)	45.5(5)
C5B	9272(4)	8495(2)	8876.1(19)	52.1(6)
C6A	2097(3)	4987(2)	5966(2)	49.5(6)
C6B	10244(4)	7950(3)	9700(2)	58.8(7)
C7A	2740(3)	6016(2)	4370(2)	47.4(6)
C7B	10285(4)	9538(2)	8454(2)	52.4(6)
C8A	2863(3)	4858(2)	4235(2)	52.1(6)
C8B	11873(4)	9584(3)	9011(2)	58.8(7)
C9A	2475(4)	4161(2)	5218(2)	54.5(7)
C9B	11938(4)	8591(3)	9802(2)	63.2(7)
C10A	3000(4)	6834(2)	3539(2)	56.6(7)
C10B	9907(5)	10413(3)	7691(2)	61.6(7)
C11A	3365(4)	6473(3)	2595(2)	65.9(8)
C11B	11136(5)	11291(3)	7490(2)	68.9(8)
C12A	3496(5)	5328(3)	2481(3)	71.9(9)
C12B	12721(5)	11310(3)	8018(3)	75.9(9)
C13A	3241(4)	4509(3)	3300(3)	65.0(8)
C13B	13101(5)	10450(3)	8801(3)	72.4(9)
C14A	2418(3)	8227(2)	5580(2)	48.6(6)
C14B	6736(4)	8376(3)	7674(2)	57.8(7)

Table S5. Anisotropic Displacement Parameters (Å²×10³) for **KFI** (CCDC 2380849). The anisotropic displacement factor exponent takes the form: $-2\pi^2[h^2a^{*2}U_{11} + 2hkab^{*}U_{12} + \ldots]$.

Atom	U_{11}	U_{22}	U_{33}	U_{23}	U_{13}	U_{12}
K1	71.7(5)	59.9(5)	75.9(5)	3.3(3)	3.7(3)	-4.6(3)
K2	77.7(5)	55.7(4)	67.6(4)	1.9(3)	10.0(3)	2.6(3)
O1	97(2)	81.4(19)	108(2)	-11.7(15)	-4.4(17)	-21.0(16)
O2	84.2(17)	79.2(19)	93.0(18)	-4.6(14)	13.6(13)	-28.1(14)
O3	79.5(16)	73.0(16)	81.0(16)	5.1(12)	4.1(12)	23.3(13)
O4	73.9(15)	68.5(16)	82.4(16)	-6.7(12)	9.7(13)	15.0(12)
O5	118(2)	69.7(16)	74.6(16)	11.2(12)	13.5(15)	18.2(15)
C1	77(2)	64(2)	68(2)	-8.3(16)	16.3(16)	-13.8(17)
C2	67.3(19)	60.1(19)	57.1(15)	-1.8(13)	7.0(13)	-9.7(15)
C3	66.9(18)	56.2(18)	54.7(15)	-1.5(13)	2.5(13)	-5.0(15)
C4	67.9(18)	54.2(18)	59.0(15)	-0.1(12)	4.8(13)	-7.5(14)
C5	69(2)	67(2)	62.4(17)	-2.3(15)	4.9(15)	-12.9(17)
C6	70(2)	62(2)	55.0(16)	-2.1(14)	-2.4(14)	6.3(16)
C7	64.6(17)	55.4(18)	52.7(14)	0.3(12)	9.5(12)	6.0(14)
C8	64.1(17)	51.7(17)	51.4(14)	3.3(12)	10.4(12)	4.1(14)
C9	66.4(18)	53.7(17)	53.5(14)	4.4(12)	12.6(13)	9.2(14)
C10	67.4(19)	69(2)	57.8(16)	7.4(14)	11.3(13)	12.0(16)
C11	85(3)	94(3)	81(2)	3(2)	-3(2)	-21(2)
C12	68(2)	91(3)	75(2)	6.1(19)	6.7(16)	-6(2)
C13	65.9(19)	67(2)	57.5(16)	4.6(14)	8.8(13)	4.7(16)
C14	80(2)	54.0(18)	62.4(17)	4.2(14)	14.6(15)	6.5(16)
C15	74(2)	60(2)	66.0(18)	-1.7(15)	14.2(15)	-5.2(16)
C16	63.9(18)	65(2)	65.2(18)	-1.6(15)	10.2(14)	0.5(16)
O17	188(4)	88(2)	76.2(18)	9.6(15)	6(2)	-45(2)
C18	65.8(19)	72(2)	62.6(17)	-2.0(15)	5.9(14)	-1.5(17)
C19	72(2)	62(2)	59.7(17)	-0.5(14)	3.6(14)	-2.0(16)
C20	84(2)	53.1(19)	73(2)	1.5(15)	8.6(16)	-6.6(17)
C21	85(2)	62(2)	77(2)	-0.6(16)	13.0(18)	9.4(18)
C22	66(2)	70(2)	76(2)	2.0(16)	8.1(16)	2.9(17)
C23	96(3)	58(2)	87(2)	-4.2(17)	14(2)	-3.1(19)
C24	113(4)	76(3)	100(3)	-4(2)	17(3)	17(3)
C25	88(3)	104(4)	97(3)	-9(2)	8(2)	27(3)
C26	74(2)	97(3)	85(2)	-2(2)	3.4(18)	1(2)
C27	69.1(19)	61.5(19)	51.0(15)	3.8(13)	6.6(13)	1.8(15)
C28	82(2)	63(2)	68.8(19)	-0.6(15)	0.5(17)	1.6(18)
C29	115(3)	66(2)	84(2)	0.0(19)	-7(2)	-15(2)

Table S6. Anisotropic Displacement Parameters (Å²×10³) for **HKFI₂** (CCDC 2345911). The anisotropic displacement factor exponent takes the form: $-2\pi^2[h^2a^{*2}U_{11} + 2hkab^*U_{12} + \ldots]$.

Atom	U_{11}	U_{22}	U_{33}	U_{23}	U_{13}	U_{12}
K1	56.4(4)	43.1(3)	68.4(4)	-5.7(2)	-17.1(3)	-4.8(2)
O1A	73.8(14)	38.8(10)	105.5(17)	-9.1(10)	-17.0(12)	-8.3(9)
O1B	74.1(15)	110.4(19)	73.4(15)	3.4(13)	-34.9(12)	-8.9(13)
O2A	60.3(11)	43.7(9)	77.3(13)	-3.1(9)	-26.9(10)	-1.1(8)
O2B	82.4(16)	121(2)	61.4(14)	5.5(13)	-25.9(12)	-39.5(15)
O3A	60.1(11)	48.0(10)	85.7(14)	-5.0(9)	-32.0(10)	-11.3(8)
O3B	57.0(12)	77.6(14)	72.2(14)	-5.1(11)	-19.2(10)	-11.2(10)
C1A	53.5(15)	51.0(15)	70.9(19)	6.3(13)	-8.5(13)	-9.7(12)
C1B	77(2)	73(2)	60.1(18)	6.9(15)	-15.6(15)	-6.3(16)
C2A	60.9(17)	65.9(18)	56.3(17)	-0.4(13)	-5.0(13)	-8.7(13)
C2B	88(2)	73(2)	71(2)	9.6(17)	-9.6(18)	-19.3(18)
C3A	47.4(14)	55.5(15)	67.7(18)	-11.3(13)	-8.6(12)	-2.3(11)
C3B	66.6(19)	69.7(19)	67.6(19)	-4.8(15)	-8.2(15)	-17.0(15)
C4A	38.9(12)	42.2(12)	60.1(16)	-6.1(11)	-10.9(11)	-2.1(9)
C4B	56.5(15)	53.8(15)	55.1(16)	-5.5(12)	-8.2(12)	-5.7(12)
C5A	36.7(12)	40.0(12)	60.8(15)	-2.7(10)	-13.3(10)	-4.1(9)
C5B	56.2(15)	55.7(14)	45.2(14)	-7.3(11)	-7.0(11)	-4.7(12)
C6A	41.5(13)	40.5(12)	67.3(17)	-4.3(11)	-11.5(11)	-4.9(9)
C6B	61.3(16)	65.4(17)	48.7(15)	-4.2(13)	-10.1(12)	1.8(13)
C7A	39.0(12)	45.5(13)	59.3(15)	-5.3(11)	-13.1(11)	-4.1(9)
C7B	53.4(14)	56.9(15)	48.4(14)	-10.8(11)	-6.5(11)	-3.4(11)
C8A	39.7(13)	47.9(13)	71.3(18)	-10.9(12)	-15.0(12)	-2.8(10)
C8B	60.1(16)	63.4(17)	55.4(16)	-12.1(13)	-11.0(13)	-4.6(13)
C9A	42.2(13)	40.1(12)	84.1(19)	-8.8(12)	-17.6(13)	-5.2(10)
C9B	57.2(16)	80(2)	55.0(16)	-11.9(14)	-14.2(13)	-2.7(14)
C10A	53.6(15)	51.6(14)	66.4(18)	-6.5(13)	-13.6(13)	-7.0(11)
C10B	68.7(18)	60.2(16)	57.4(17)	-5.1(13)	-13.5(14)	-7.7(13)
C11A	59.5(17)	78(2)	61.5(18)	-4.0(15)	-12.7(14)	-11.0(14)
C11B	81(2)	60.1(17)	66(2)	-2.9(15)	-8.3(16)	-13.7(15)
C12A	66.0(19)	89(2)	67(2)	-26.1(18)	-12.9(15)	-6.8(16)
C12B	79(2)	71(2)	82(2)	-10.9(17)	-4.7(18)	-23.3(17)
C13A	56.8(16)	61.8(17)	82(2)	-24.0(16)	-12.7(15)	-4.9(13)
C13B	64.1(19)	79(2)	80(2)	-16.7(17)	-18.2(16)	-15.9(16)
C14A	41.0(12)	43.8(12)	62.4(16)	-8.2(11)	-11.8(11)	-0.9(10)
C14B	57.7(16)	60.2(16)	58.1(17)	-10.7(13)	-11.3(13)	-7.8(13)

Table S7. Bond Lengths for **KFI** (CCDC 2380849).

Atom	Atom	Length/Å	Atom	Atom	Length/Å
K1	K1[1]	4.3816(9)	C1	K2[14]	3.412(4)
K1	K1[2]	4.3816(9)	C1	C2	1.513(5)
K1	K2[3]	3.8273(11)	C1	O17	1.244(5)
K1	K2[4]	4.2988(10)	C2	C3	1.392(5)
K1	O1[5]	2.746(4)	C2	C22	1.389(5)
K1	O1[6]	2.906(3)	C3	C4	1.415(4)
K1	O2	2.695(3)	C3	C19	1.476(5)
K1	O4	2.670(3)	C4	C5	1.475(5)
K1	C1[6]	3.203(4)	C4	C20	1.369(5)
K1	C6	3.500(4)	C5	C18	1.488(5)
K1	O17[6]	2.857(3)	C6	K2[3]	3.165(4)
K1	O17[7]	2.717(4)	C6	C7	1.523(4)
K2	K1[8]	3.8272(11)	C7	C8	1.381(4)
K2	K1[4]	4.2988(10)	C7	C16	1.388(5)
K2	K2[9]	4.3125(8)	C8	C9	1.409(4)
K2	K2[10]	4.3124(8)	C8	C27	1.481(4)
K2	O1[11]	2.758(3)	C9	C10	1.485(5)
K2	O3	2.694(3)	C9	C14	1.381(5)
K2	O4[4]	2.754(3)	C10	C13	1.500(5)
K2	O4[8]	2.874(3)	C11	C12	1.375(6)
K2	O5[12]	2.737(3)	C11	C29	1.385(6)
K2	O5[8]	2.805(3)	C12	C13	1.371(5)
K2	C1[11]	3.411(4)	C13	C27	1.399(4)
K2	C6[8]	3.165(4)	C14	C15	1.387(5)
O1	K1[13]	2.906(3)	C15	C16	1.385(5)
O1	K1[5]	2.746(4)	O17	K1[13]	2.857(3)
O1	K2[14]	2.758(3)	O17	K1[7]	2.717(4)
O1	C1	1.233(4)	C18	C19	1.402(5)
O2	C5	1.218(4)	C18	C26	1.372(5)
O3	C10	1.217(4)	C19	C23	1.387(5)
O4	K2[3]	2.874(3)	C20	C21	1.394(5)
O4	K2[4]	2.754(3)	C21	C22	1.399(5)
O4	C6	1.230(4)	C23	C24	1.388(6)
O5	K2[12]	2.737(3)	C24	C25	1.363(7)
O5	K2[3]	2.805(3)	C25	C26	1.377(6)
O5	C6	1.261(4)	C27	C28	1.390(5)
C1	K1[13]	3.203(4)	C28	C29	1.387(5)

[1]+X,1/2-Y,1/2+Z; [2]+X,1/2-Y,-1/2+Z; [3]-X,-1/2+Y,3/2-Z; [4]-X,1-Y,1-Z; [5]1-X,1-Y,2-Z; [6]1-X,-1/2+Y,3/2-Z; [7]1-X,1-Y,1-Z; [8]-X,1/2+Y,3/2-Z; [9]+X,3/2-Y,-1/2+Z; [10]+X,3/2-Y,1/2+Z; [11]-1+X,+Y,+Z; [12]-X,1-Y,2-Z; [13]1-X,1/2+Y,3/2-Z; [14]1+X,+Y,+Z

Table S8. Bond Lengths for **HKFI₂** (CCDC 2345911).

Atom	Atom	Length/Å	Atom	Atom	Length/Å
K1	K1[1]	4.2423(11)	C4A	C5A	1.393(3)
K1	K1[2]	4.0104(12)	C4A	C14A	1.510(3)
K1	O1A[3]	2.7088(19)	C4B	C5B	1.397(4)
K1	O2A[4]	2.709(2)	C4B	C14B	1.485(4)
K1	O2A[2]	2.8350(19)	C5A	C6A	1.407(3)
K1	O2B	2.651(2)	C5A	C7A	1.489(4)
K1	O3A	2.945(2)	C5B	C6B	1.406(4)
K1	O3A[2]	2.8201(19)	C5B	C7B	1.495(4)
K1	C11B[1]	3.402(3)	C6A	C9A	1.491(4)
K1	C14A[2]	3.180(3)	C6B	C9B	1.486(4)
O1A	C9A	1.219(3)	C7A	C8A	1.403(3)
O1B	C9B	1.222(3)	C7A	C10A	1.386(4)
O2A	C14A	1.245(3)	C7B	C8B	1.405(4)
O2B	C14B	1.214(4)	C7B	C10B	1.383(4)
O3A	C14A	1.274(3)	C8A	C9A	1.477(4)
O3B	C14B	1.304(3)	C8A	C13A	1.376(4)
C1A	C2A	1.383(4)	C8B	C9B	1.476(4)
C1A	C6A	1.373(4)	C8B	C13B	1.377(4)
C1B	C2B	1.378(5)	C10A	C11A	1.392(4)
C1B	C6B	1.371(4)	C10B	C11B	1.388(4)
C2A	C3A	1.384(4)	C11A	C12A	1.381(5)
C2B	C3B	1.391(4)	C11B	C12B	1.373(5)
C3A	C4A	1.397(4)	C12A	C13A	1.376(5)
C3B	C4B	1.397(4)	C12B	C13B	1.390(5)

[1]2-X,2-Y,1-Z; [2]1-X,2-Y,1-Z; [3]1-X,1-Y,1-Z; [4]1+X,+Y,+Z

Table S9. Bond Angles for **KFI** (CCDC 2380849).

Atom	Atom	Atom	Angle/°	Atom	Atom	Atom	Angle/°
K1^{1}	K1	K1^{2}	107.76(3)	O5^{8}	K2	K2^{10}	38.35(6)
K2^{3}	K1	K1^{2}	62.73(2)	O5^{12}	K2	K2^{10}	138.94(7)
K2^{3}	K1	K1^{1}	71.39(2)	O5^{12}	K2	K2^{9}	39.48(5)
K2^{4}	K1	K1^{2}	126.51(2)	O5^{12}	K2	O1^{11}	86.90(10)
K2^{4}	K1	K1^{1}	52.313(13)	O5^{12}	K2	O4^{4}	162.74(9)
K2^{3}	K1	K2^{4}	63.795(19)	O5^{8}	K2	O4^{8}	46.00(8)
O1^{5}	K1	K1^{2}	40.52(6)	O5^{12}	K2	O4^{8}	75.76(8)
O1^{6}	K1	K1^{1}	37.88(7)	O5^{12}	K2	O5^{8}	100.60(9)
O1^{5}	K1	K1^{1}	146.88(7)	O5^{12}	K2	C1^{11}	105.59(9)
O1^{6}	K1	K1^{2}	70.63(7)	O5^{8}	K2	C1^{11}	123.37(10)
O1^{6}	K1	K2^{4}	72.05(6)	O5^{12}	K2	C6^{8}	85.90(8)
O1^{5}	K1	K2^{4}	130.62(7)	O5^{8}	K2	C6^{8}	23.39(8)
O1^{6}	K1	K2^{3}	45.88(7)	C1^{11}	K2	K1^{8}	52.15(7)
O1^{5}	K1	K2^{3}	81.89(6)	C1^{11}	K2	K1^{4}	54.78(6)
O1^{5}	K1	O1^{6}	109.02(9)	C1^{11}	K2	K2^{10}	101.01(7)
O1^{5}	K1	C1^{6}	95.12(9)	C1^{11}	K2	K2^{9}	91.43(6)
O1^{6}	K1	C1^{6}	22.61(9)	C6^{8}	K2	K1^{8}	59.18(7)
O1^{5}	K1	C6	75.86(9)	C6^{8}	K2	K1^{4}	97.21(6)
O1^{6}	K1	C6	93.82(9)	C6^{8}	K2	K2^{10}	55.18(6)
O1^{5}	K1	O17^{6}	76.28(9)	C6^{8}	K2	K2^{9}	56.45(6)
O2	K1	K1^{1}	122.21(6)	C6^{8}	K2	C1^{11}	111.32(9)
O2	K1	K1^{2}	127.64(6)	K1^{5}	O1	K1^{13}	101.60(10)
O2	K1	K2^{3}	145.22(7)	K1^{5}	O1	K2^{14}	121.86(11)
O2	K1	K2^{4}	97.80(6)	K2^{14}	O1	K1^{13}	84.98(9)
O2	K1	O1^{5}	90.89(9)	C1	O1	K1^{13}	92.4(3)
O2	K1	O1^{6}	159.82(10)	C1	O1	K1^{5}	125.4(3)
O2	K1	C1^{6}	157.53(10)	C1	O1	K2^{14}	111.7(3)
O2	K1	C6	94.29(9)	C5	O2	K1	152.3(3)
O2	K1	O17^{6}	143.45(11)	C10	O3	K2	149.7(3)
O2	K1	O17^{7}	83.90(9)	K1	O4	K2^{3}	87.23(7)
O4	K1	K1^{1}	84.48(6)	K1	O4	K2^{4}	104.84(9)
O4	K1	K1^{2}	101.24(6)	K2^{4}	O4	K2^{3}	100.00(8)
O4	K1	K2^{4}	38.27(6)	C6	O4	K1	123.4(2)
O4	K1	K2^{3}	48.60(6)	C6	O4	K2^{3}	92.0(2)
O4	K1	O1^{5}	92.44(9)	C6	O4	K2^{4}	130.8(2)
O4	K1	O1^{6}	84.59(8)	K2^{12}	O5	K2^{3}	102.17(9)
O4	K1	O2	98.23(8)	C6	O5	K2^{3}	94.6(2)
O4	K1	C1^{6}	103.11(9)	C6	O5	K2^{12}	124.9(2)
O4	K1	C6	17.07(7)	K1^{13}	C1	K2^{14}	70.62(7)
O4	K1	O17^{6}	116.13(11)	O1	C1	K1^{13}	65.0(2)
O4	K1	O17^{7}	98.72(11)	O1	C1	K2^{14}	48.7(2)

Atom	Atom	Atom	Angle/°
C1^{6}	K1	K1^{2}	54.61(7)
C1^{6}	K1	K1^{1}	54.06(7)
C1^{6}	K1	K2^{4}	94.64(7)
C1^{6}	K1	K2^{3}	57.23(7)
C1^{6}	K1	C6	108.18(9)
C6	K1	K1^{1}	100.57(5)
C6	K1	K1^{2}	90.86(6)
C6	K1	K2^{4}	55.14(5)
C6	K1	K2^{3}	50.94(6)
O17^{6}	K1	K1^{1}	75.63(7)
O17^{7}	K1	K1^{2}	138.85(8)
O17^{6}	K1	K1^{2}	37.06(7)
O17^{7}	K1	K1^{1}	39.33(6)
O17^{6}	K1	K2^{3}	67.53(9)
O17^{6}	K1	K2^{4}	116.65(8)
O17^{7}	K1	K2^{4}	60.77(9)
O17^{7}	K1	K2^{3}	108.30(7)
O17^{7}	K1	O1^{6}	75.92(9)
O17^{7}	K1	O1^{5}	168.24(11)
O17^{6}	K1	O1^{6}	44.90(9)
O17^{7}	K1	C1^{6}	85.98(9)
O17^{6}	K1	C1^{6}	22.78(9)
O17^{6}	K1	C6	114.75(11)
O17^{7}	K1	C6	114.96(11)
O17^{7}	K1	O17^{6}	101.80(11)
K1^{8}	K2	K1^{4}	64.957(19)
K1^{4}	K2	K2^{9}	128.37(2)
K1^{4}	K2	K2^{10}	52.776(13)
K1^{8}	K2	K2^{10}	72.18(2)
K1^{8}	K2	K2^{9}	63.43(2)
K2^{9}	K2	K2^{10}	110.32(3)
O1^{11}	K2	K1^{8}	49.15(7)
O1^{11}	K2	K1^{4}	73.11(7)
O1^{11}	K2	K2^{10}	113.12(7)
O1^{11}	K2	K2^{9}	72.94(6)
O1^{11}	K2	O4^{8}	83.63(8)
O1^{11}	K2	O5^{8}	123.25(9)
O1^{11}	K2	C1^{11}	19.62(9)
O1^{11}	K2	C6^{8}	104.69(9)
O3	K2	K1^{4}	95.96(6)
O3	K2	K1^{8}	143.26(6)
O3	K2	K2^{9}	125.50(6)

Atom	Atom	Atom	Angle/°
O1	C1	C2	116.7(3)
O1	C1	O17	125.5(4)
C2	C1	K1^{13}	162.8(2)
C2	C1	K2^{14}	124.4(2)
O17	C1	K1^{13}	62.8(2)
O17	C1	K2^{14}	98.6(3)
O17	C1	C2	117.8(3)
C3	C2	C1	122.7(3)
C22	C2	C1	119.9(3)
C22	C2	C3	117.3(3)
C2	C3	C4	120.3(3)
C2	C3	C19	131.7(3)
C4	C3	C19	108.0(3)
C3	C4	C5	108.7(3)
C20	C4	C3	121.7(3)
C20	C4	C5	129.5(3)
O2	C5	C4	128.0(3)
O2	C5	C18	126.0(3)
C4	C5	C18	106.0(3)
K2^{3}	C6	K1	69.88(7)
O4	C6	K1	39.57(16)
O4	C6	K2^{3}	65.18(19)
O4	C6	O5	126.1(3)
O4	C6	C7	117.9(3)
O5	C6	K1	109.0(2)
O5	C6	K2^{3}	62.06(19)
O5	C6	C7	115.9(3)
C7	C6	K1	121.7(2)
C7	C6	K2^{3}	166.3(2)
C8	C7	C6	121.6(3)
C8	C7	C16	117.9(3)
C16	C7	C6	120.4(3)
C7	C8	C9	120.0(3)
C7	C8	C27	131.8(3)
C9	C8	C27	108.2(3)
C8	C9	C10	108.8(3)
C14	C9	C8	121.8(3)
C14	C9	C10	129.3(3)
O3	C10	C9	127.9(3)
O3	C10	C13	126.4(3)
C9	C10	C13	105.7(3)
C12	C11	C29	120.4(4)

Atom	Atom	Atom	Angle/°	Atom	Atom	Atom	Angle/°
O3	K2	K2^{10}	122.47(6)	C13	C12	C11	118.2(4)
O3	K2	O1^{11}	96.48(9)	C12	C13	C10	129.1(3)
O3	K2	O4^{8}	163.68(8)	C12	C13	C27	122.5(3)
O3	K2	O4^{4}	83.75(8)	C27	C13	C10	108.2(3)
O3	K2	O5^{12}	87.93(8)	C9	C14	C15	117.6(3)
O3	K2	O5^{8}	139.51(9)	C16	C15	C14	120.7(3)
O3	K2	C1^{11}	91.12(9)	C15	C16	C7	121.9(3)
O3	K2	C6^{8}	157.56(9)	K1^{7}	O17	K1^{13}	103.61(10)
O4^{4}	K2	K1^{8}	95.02(5)	C1	O17	K1^{7}	129.2(3)
O4^{4}	K2	K1^{4}	36.90(6)	C1	O17	K1^{13}	94.4(2)
O4^{8}	K2	K1^{4}	99.66(6)	C19	C18	C5	108.5(3)
O4^{8}	K2	K1^{8}	44.17(5)	C26	C18	C5	129.4(4)
O4^{4}	K2	K2^{10}	41.03(5)	C26	C18	C19	122.1(4)
O4^{8}	K2	K2^{10}	71.66(6)	C18	C19	C3	108.8(3)
O4^{8}	K2	K2^{9}	38.97(6)	C23	C19	C3	132.3(3)
O4^{4}	K2	K2^{9}	150.69(6)	C23	C19	C18	118.8(3)
O4^{4}	K2	O1^{11}	109.01(9)	C4	C20	C21	118.3(3)
O4^{4}	K2	O4^{8}	111.74(7)	C20	C21	C22	120.0(3)
O4^{4}	K2	O5^{8}	76.64(8)	C2	C22	C21	122.2(3)
O4^{8}	K2	C1^{11}	94.01(8)	C19	C23	C24	117.7(4)
O4^{4}	K2	C1^{11}	89.71(8)	C25	C24	C23	123.1(4)
O4^{8}	K2	C6^{8}	22.85(8)	C24	C25	C26	119.6(4)
O4^{4}	K2	C6^{8}	96.08(8)	C18	C26	C25	118.7(4)
O5^{8}	K2	K1^{4}	89.21(6)	C13	C27	C8	109.1(3)
O5^{12}	K2	K1^{4}	159.93(7)	C28	C27	C8	131.9(3)
O5^{12}	K2	K1^{8}	100.65(6)	C28	C27	C13	118.9(3)
O5^{8}	K2	K1^{8}	74.33(7)	C29	C28	C27	118.3(4)
O5^{8}	K2	K2^{9}	78.35(6)	C11	C29	C28	121.7(4)

^{1}+X,1/2-Y,-1/2+Z; ^{2}+X,1/2-Y,1/2+Z; ^{3}-X,-1/2+Y,3/2-Z; ^{4}-X,1-Y,1-Z; ^{5}1-X,1-Y,2-Z; ^{6}1-X,-1/2+Y,3/2-Z; ^{7}1-X,1-Y,1-Z; ^{8}-X,1/2+Y,3/2-Z; ^{9}+X,3/2-Y,1/2+Z; ^{10}+X,3/2-Y,-1/2+Z; ^{11}-1+X,+Y,+Z; ^{12}-X,1-Y,2-Z; ^{13}1-X,1/2+Y,3/2-Z; ^{14}1+X,+Y,+Z

Table S10. Bond Angles for **HKFI$_2$** (CCDC 2345911).

Atom	Atom	Atom	Angle/°	Atom	Atom	Atom	Angle/°
K1^{1}	K1	K1^{2}	115.28(3)	C2A	C3A	C4A	122.2(3)
O1A^{3}	K1	K1^{1}	116.85(5)	C2B	C3B	C4B	122.3(3)
O1A^{3}	K1	K1^{2}	127.50(5)	C3A	C4A	C14A	118.3(2)
O1A^{3}	K1	O2A^{1}	165.90(7)	C5A	C4A	C3A	117.9(2)
O1A^{3}	K1	O2A^{4}	86.47(6)	C5A	C4A	C14A	123.9(2)
O1A^{3}	K1	O3A^{1}	138.64(7)	C3B	C4B	C14B	118.4(3)
O1A^{3}	K1	O3A	84.05(6)	C5B	C4B	C3B	118.2(3)
O1A^{3}	K1	C11B^{2}	67.29(8)	C5B	C4B	C14B	123.1(3)
O1A^{3}	K1	C14A^{1}	157.74(8)	C4A	C5A	C6A	118.7(2)
O2A^{1}	K1	K1^{1}	76.30(4)	C4A	C5A	C7A	133.0(2)
O2A^{4}	K1	K1^{2}	41.18(4)	C6A	C5A	C7A	108.3(2)
O2A^{1}	K1	K1^{2}	38.99(4)	C4B	C5B	C6B	118.3(3)
O2A^{4}	K1	K1^{1}	156.44(5)	C4B	C5B	C7B	133.9(2)
O2A^{4}	K1	O2A^{1}	80.18(6)	C6B	C5B	C7B	107.8(2)
O2A^{1}	K1	O3A	109.87(6)	C1A	C6A	C5A	122.9(3)
O2A^{4}	K1	O3A^{1}	113.12(6)	C1A	C6A	C9A	128.6(2)
O2A^{4}	K1	O3A	150.72(6)	C5A	C6A	C9A	108.5(2)
O2A^{1}	K1	C11B^{2}	104.81(7)	C1B	C6B	C5B	122.6(3)
O2A^{4}	K1	C11B^{2}	76.43(7)	C1B	C6B	C9B	128.5(3)
O2A^{4}	K1	C14A^{1}	94.87(6)	C5B	C6B	C9B	108.9(3)
O2A^{1}	K1	C14A^{1}	22.98(6)	C8A	C7A	C5A	108.1(2)
O2B	K1	K1^{2}	82.36(5)	C10A	C7A	C5A	132.6(2)
O2B	K1	K1^{1}	92.11(7)	C10A	C7A	C8A	119.2(3)
O2B	K1	O1A^{3}	101.39(8)	C8B	C7B	C5B	108.3(2)
O2B	K1	O2A^{1}	82.31(7)	C10B	C7B	C5B	132.9(3)
O2B	K1	O2A^{4}	86.08(7)	C10B	C7B	C8B	118.7(3)
O2B	K1	O3A^{1}	115.42(8)	C7A	C8A	C9A	109.2(2)
O2B	K1	O3A	68.84(7)	C13A	C8A	C7A	121.9(3)
O2B	K1	C11B^{2}	159.47(9)	C13A	C8A	C9A	128.9(3)
O2B	K1	C14A^{1}	100.87(8)	C7B	C8B	C9B	108.9(3)
O3A	K1	K1^{1}	44.65(4)	C13B	C8B	C7B	122.2(3)
O3A	K1	K1^{2}	141.64(5)	C13B	C8B	C9B	128.9(3)
O3A^{1}	K1	K1^{1}	47.22(5)	O1A	C9A	C6A	126.8(3)
O3A^{1}	K1	K1^{2}	77.79(4)	O1A	C9A	C8A	127.3(3)
O3A^{1}	K1	O2A^{1}	46.23(5)	C8A	C9A	C6A	105.8(2)
O3A^{1}	K1	O3A	91.87(6)	O1B	C9B	C6B	126.5(3)
O3A	K1	C11B^{2}	124.17(7)	O1B	C9B	C8B	127.4(3)
O3A^{1}	K1	C11B^{2}	81.85(7)	C8B	C9B	C6B	106.1(2)
O3A^{1}	K1	C14A^{1}	23.55(5)	C7A	C10A	C11A	118.5(3)
O3A	K1	C14A^{1}	104.26(6)	C7B	C10B	C11B	118.8(3)
C11B^{2}	K1	K1^{2}	91.20(6)	C12A	C11A	C10A	121.3(3)

Atom	Atom	Atom	Angle/°	Atom	Atom	Atom	Angle/°
C11B[2]	K1	K1[1]	108.22(6)	C10B	C11B	K1[2]	96.4(2)
C14A[1]	K1	K1[2]	55.87(4)	C12B	C11B	K1[2]	111.5(2)
C14A[1]	K1	K1[1]	62.37(5)	C12B	C11B	C10B	122.0(3)
C14A[1]	K1	C11B[2]	91.38(8)	C13A	C12A	C11A	120.7(3)
C9A	O1A	K1[3]	159.6(2)	C11B	C12B	C13B	120.0(3)
K1[5]	O2A	K1[1]	99.82(6)	C8A	C13A	C12A	118.4(3)
C14A	O2A	K1[5]	127.25(18)	C8B	C13B	C12B	118.2(3)
C14A	O2A	K1[1]	94.27(15)	O2A	C14A	K1[1]	62.75(13)
C14B	O2B	K1	139.4(2)	O2A	C14A	O3A	123.6(2)
K1[1]	O3A	K1	88.13(6)	O2A	C14A	C4A	119.6(2)
C14A	O3A	K1[1]	94.28(14)	O3A	C14A	K1[1]	62.17(13)
C14A	O3A	K1	122.86(18)	O3A	C14A	C4A	116.8(2)
C6A	C1A	C2A	118.1(3)	C4A	C14A	K1[1]	167.57(18)
C6B	C1B	C2B	119.2(3)	O2B	C14B	O3B	122.8(3)
C1A	C2A	C3A	120.1(3)	O2B	C14B	C4B	122.1(3)
C1B	C2B	C3B	119.2(3)	O3B	C14B	C4B	114.8(3)

[1]1-X,2-Y,1-Z; [2]2-X,2-Y,1-Z; [3]1-X,1-Y,1-Z; [4]1+X,+Y,+Z; [5]-1+X,+Y,+Z

Table S11. Torsion Angles for **KFI** (CCDC 2380849).

A	B	C	D	Angle/°
K1^{1}	O1	C1	K1^{2}	106.0(3)
K1^{1}	O1	C1	K2^{3}	-168.4(4)
K1^{2}	O1	C1	K2^{3}	85.54(16)
K1^{2}	O1	C1	C2	-160.9(3)
K1^{1}	O1	C1	C2	-54.9(4)
K1^{1}	O1	C1	O17	124.0(4)
K1^{2}	O1	C1	O17	18.0(5)
K1	O2	C5	C4	-2.4(7)
K1	O2	C5	C18	179.7(3)
K1	O4	C6	K2^{4}	-87.98(19)
K1	O4	C6	O5	-75.6(4)
K1	O4	C6	C7	107.0(3)
K1^{2}	C1	C2	C3	16.9(10)
K1^{2}	C1	C2	C22	-165.7(7)
K1^{2}	C1	O17	K1^{5}	111.6(3)
K1	C6	C7	C8	150.8(2)
K1	C6	C7	C16	-32.3(4)
K2^{3}	O1	C1	K1^{2}	-85.54(16)
K2^{3}	O1	C1	C2	113.5(3)
K2^{3}	O1	C1	O17	-67.6(5)
K2	O3	C10	C9	5.9(7)
K2	O3	C10	C13	-174.0(3)
K2^{4}	O4	C6	K1	87.98(19)
K2^{6}	O4	C6	K1	-167.0(4)
K2^{6}	O4	C6	K2^{4}	105.0(3)
K2^{4}	O4	C6	O5	12.4(4)
K2^{6}	O4	C6	O5	117.5(3)
K2^{4}	O4	C6	C7	-165.0(2)
K2^{6}	O4	C6	C7	-60.0(4)
K2^{7}	O5	C6	K1	54.7(3)
K2^{4}	O5	C6	K1	-53.51(16)
K2^{7}	O5	C6	K2^{4}	108.2(2)
K2^{7}	O5	C6	O4	95.5(4)
K2^{4}	O5	C6	O4	-12.8(4)
K2^{4}	O5	C6	C7	164.7(2)
K2^{7}	O5	C6	C7	-87.0(3)
K2^{3}	C1	C2	C3	165.0(2)
K2^{3}	C1	C2	C22	-17.6(4)
K2^{3}	C1	O17	K1^{5}	48.7(3)
K2^{3}	C1	O17	K1^{2}	-62.94(16)
K2^{4}	C6	C7	C8	5.5(11)
K2^{4}	C6	C7	C16	-177.6(8)
O1	C1	C2	C3	108.4(4)

A	B	C	D	Angle/°
C3	C4	C5	O2	-179.4(3)
C3	C4	C5	C18	-1.1(3)
C3	C4	C20	C21	2.2(5)
C3	C19	C23	C24	-177.5(4)
C4	C3	C19	C18	-2.2(3)
C4	C3	C19	C23	174.3(4)
C4	C5	C18	C19	-0.3(4)
C4	C5	C18	C26	-177.7(3)
C4	C20	C21	C22	-1.8(5)
C5	C4	C20	C21	179.8(3)
C5	C18	C19	C3	1.5(4)
C5	C18	C19	C23	-175.6(3)
C5	C18	C26	C25	176.2(4)
C6	C7	C8	C9	174.7(3)
C6	C7	C8	C27	-4.8(5)
C6	C7	C16	C15	-174.6(3)
C7	C8	C9	C10	-177.4(3)
C7	C8	C9	C14	0.2(5)
C7	C8	C27	C13	177.0(3)
C7	C8	C27	C28	-6.1(6)
C8	C7	C16	C15	2.4(5)
C8	C9	C10	O3	178.9(3)
C8	C9	C10	C13	-1.2(3)
C8	C9	C14	C15	1.7(5)
C8	C27	C28	C29	-177.1(3)
C9	C8	C27	C13	-2.5(3)
C9	C8	C27	C28	174.3(3)
C9	C10	C13	C12	-175.8(3)
C9	C10	C13	C27	-0.3(3)
C9	C14	C15	C16	-1.6(5)
C10	C9	C14	C15	178.7(3)
C10	C13	C27	C8	1.7(3)
C10	C13	C27	C28	-175.6(3)
C11	C12	C13	C10	175.0(4)
C11	C12	C13	C27	0.1(5)
C12	C11	C29	C28	-0.1(7)
C12	C13	C27	C8	177.6(3)
C12	C13	C27	C28	0.2(5)
C13	C27	C28	C29	-0.5(5)
C14	C9	C10	O3	1.5(6)
C14	C9	C10	C13	-178.6(3)
C14	C15	C16	C7	-0.5(5)
C16	C7	C8	C9	-2.3(4)

A	B	C	D	Angle/°	A	B	C	D	Angle/°
O1	C1	C2	C22	-74.1(4)	C16	C7	C8	C27	178.2(3)
O1	C1	O17	K1[2]	-18.3(5)	O17	C1	C2	C3	-70.6(5)
O1	C1	O17	K1[5]	93.2(5)	O17	C1	C2	C22	106.9(4)
O2	C5	C18	C19	178.1(3)	C18	C19	C23	C24	-1.3(5)
O2	C5	C18	C26	0.7(6)	C19	C3	C4	C5	2.0(3)
O3	C10	C13	C12	4.1(6)	C19	C3	C4	C20	180.0(3)
O3	C10	C13	C27	179.6(3)	C19	C18	C26	C25	-0.9(6)
O4	C6	C7	C8	105.1(4)	C19	C23	C24	C25	-0.4(7)
O4	C6	C7	C16	-78.0(4)	C20	C4	C5	O2	2.8(6)
O5	C6	C7	C8	-72.6(4)	C20	C4	C5	C18	-178.9(3)
O5	C6	C7	C16	104.3(4)	C20	C21	C22	C2	-0.3(5)
C1	C2	C3	C4	175.8(3)	C22	C2	C3	C4	-1.7(4)
C1	C2	C3	C19	-4.7(5)	C22	C2	C3	C19	177.8(3)
C1	C2	C22	C21	-175.6(3)	C23	C24	C25	C26	1.6(7)
C2	C1	O17	K1[5]	-87.9(4)	C24	C25	C26	C18	-0.9(7)
C2	C1	O17	K1[2]	160.6(3)	C26	C18	C19	C3	179.1(3)
C2	C3	C4	C5	-178.5(3)	C26	C18	C19	C23	2.1(5)
C2	C3	C4	C20	-0.5(5)	C27	C8	C9	C10	2.3(3)
C2	C3	C19	C18	178.4(3)	C27	C8	C9	C14	179.9(3)
C2	C3	C19	C23	-5.2(6)	C27	C28	C29	C11	0.4(6)
C3	C2	C22	C21	2.0(5)	C29	C11	C12	C13	-0.2(6)

[1]1-X,1-Y,2-Z; [2]1-X,1/2+Y,3/2-Z; [3]1+X,+Y,+Z; [4]-X,-1/2+Y,3/2-Z; [5]1-X,1-Y,1-Z; [6]-X,1-Y,1-Z; [7]-X,1-Y,2-Z

Table S12. Torsion Angles for **HKFI$_2$** (CCDC 2345911).

A	B	C	D	Angle/°	A	B	C	D	Angle/°
K1[1]	O1A	C9A	C6A	164.8(4)	C5A	C6A	C9A	C8A	-1.2(3)
K1[1]	O1A	C9A	C8A	-16.4(7)	C5A	C7A	C8A	C9A	0.8(3)
K1[2]	O2A	C14A	K1[3]	-105.77(17)	C5A	C7A	C8A	C13A	179.2(2)
K1[3]	O2A	C14A	O3A	-13.3(3)	C5A	C7A	C10A	C11A	-178.3(3)
K1[2]	O2A	C14A	O3A	-119.1(3)	C5B	C4B	C14B	O2B	24.3(5)
K1[3]	O2A	C14A	C4A	166.1(2)	C5B	C4B	C14B	O3B	-160.9(3)
K1[2]	O2A	C14A	C4A	60.3(3)	C5B	C6B	C9B	O1B	-177.2(3)
K1	O2B	C14B	O3B	-21.5(6)	C5B	C6B	C9B	C8B	2.4(3)
K1	O2B	C14B	C4B	152.9(2)	C5B	C7B	C8B	C9B	-0.1(3)
K1	O3A	C14A	K1[3]	-90.54(14)	C5B	C7B	C8B	C13B	179.8(3)
K1[3]	O3A	C14A	O2A	13.4(3)	C5B	C7B	C10B	C11B	178.7(3)
K1	O3A	C14A	O2A	-77.1(3)	C6A	C1A	C2A	C3A	-2.0(4)
K1	O3A	C14A	C4A	103.4(2)	C6A	C5A	C7A	C8A	-1.5(3)
K1[3]	O3A	C14A	C4A	-166.1(2)	C6A	C5A	C7A	C10A	177.2(3)
K1[4]	C11B	C12B	C13B	-114.8(3)	C6B	C1B	C2B	C3B	1.6(6)
C1A	C2A	C3A	C4A	2.3(4)	C6B	C5B	C7B	C8B	1.6(3)
C1A	C6A	C9A	O1A	-2.1(4)	C6B	C5B	C7B	C10B	-175.9(3)
C1A	C6A	C9A	C8A	178.9(3)	C7A	C5A	C6A	C1A	-178.4(2)
C1B	C2B	C3B	C4B	1.2(6)	C7A	C5A	C6A	C9A	1.7(3)
C1B	C6B	C9B	O1B	3.1(6)	C7A	C8A	C9A	O1A	-178.8(2)
C1B	C6B	C9B	C8B	-177.3(3)	C7A	C8A	C9A	C6A	0.2(3)
C2A	C1A	C6A	C5A	-1.0(4)	C7A	C8A	C13A	C12A	-0.2(4)
C2A	C1A	C6A	C9A	178.9(2)	C7A	C10A	C11A	C12A	-0.9(4)
C2A	C3A	C4A	C5A	0.4(4)	C7B	C5B	C6B	C1B	177.2(3)
C2A	C3A	C4A	C14A	-178.8(2)	C7B	C5B	C6B	C9B	-2.4(3)
C2B	C1B	C6B	C5B	-1.0(5)	C7B	C8B	C9B	O1B	178.3(3)
C2B	C1B	C6B	C9B	178.6(3)	C7B	C8B	C9B	C6B	-1.3(3)
C2B	C3B	C4B	C5B	-4.5(5)	C7B	C8B	C13B	C12B	0.9(5)
C2B	C3B	C4B	C14B	169.3(3)	C7B	C10B	C11B	K1[4]	121.0(3)
C3A	C4A	C5A	C6A	-3.3(3)	C7B	C10B	C11B	C12B	0.6(5)
C3A	C4A	C5A	C7A	179.4(2)	C8A	C7A	C10A	C11A	0.3(4)
C3A	C4A	C14A	K1[3]	-26.7(9)	C8B	C7B	C10B	C11B	1.4(4)
C3A	C4A	C14A	O2A	-124.1(3)	C9A	C8A	C13A	C12A	177.8(3)
C3A	C4A	C14A	O3A	55.4(3)	C9B	C8B	C13B	C12B	-179.1(3)
C3B	C4B	C5B	C6B	4.9(4)	C10A	C7A	C8A	C9A	-178.2(2)
C3B	C4B	C5B	C7B	-174.5(3)	C10A	C7A	C8A	C13A	0.2(4)
C3B	C4B	C14B	O2B	-149.1(3)	C10A	C11A	C12A	C13A	0.9(5)
C3B	C4B	C14B	O3B	25.6(4)	C10B	C7B	C8B	C9B	177.8(3)
C4A	C5A	C6A	C1A	3.7(4)	C10B	C7B	C8B	C13B	-2.3(5)
C4A	C5A	C6A	C9A	-176.2(2)	C10B	C11B	C12B	C13B	-2.0(5)
C4A	C5A	C7A	C8A	175.9(2)	C11A	C12A	C13A	C8A	-0.3(5)
C4A	C5A	C7A	C10A	-5.3(5)	C11B	C12B	C13B	C8B	1.2(5)
C4B	C5B	C6B	C1B	-2.4(5)	C13A	C8A	C9A	O1A	3.0(4)
C4B	C5B	C6B	C9B	178.0(3)	C13A	C8A	C9A	C6A	-178.1(3)
C4B	C5B	C7B	C8B	-178.9(3)	C13B	C8B	C9B	O1B	-1.7(6)
C4B	C5B	C7B	C10B	3.6(6)	C13B	C8B	C9B	C6B	178.7(3)
C5A	C4A	C14A	K1[3]	154.1(6)	C14A	C4A	C5A	C6A	175.8(2)

A	B	C	D	Angle/°	A	B	C	D	Angle/°
C5A	C4A	C14A	O2A	56.8(3)	C14A	C4A	C5A	C7A	-1.4(4)
C5A	C4A	C14A	O3A	-123.8(3)	C14B	C4B	C5B	C6B	-168.5(3)
C5A	C6A	C9A	O1A	177.8(2)	C14B	C4B	C5B	C7B	12.1(5)

[1]1-X,1-Y,1-Z; [2]-1+X,+Y,+Z; [3]1-X,2-Y,1-Z; [4]2-X,2-Y,1-Z

Table S13. Hydrogen Atom Coordinates (Å×10^4) and Isotropic Displacement Parameters (Å^2×10^3) for **KFI** (CCDC 2380849).

Atom	*x*	*y*	*z*	U(eq)
H11	-2084	2962	4386	105
H12	-2130	4158	5044	93
H14	130	6050	8108	78
H15	1414	5873	9268	79
H16	1942	4760	9369	77
H20	5205	3957	7249	84
H21	6498	4114	6566	89
H22	7004	5240	6349	85
H23	5112	7182	7854	96
H24	3984	7720	8699	115
H25	2882	7098	9126	115
H26	2886	5878	8847	103
H28	131	2863	6588	86
H29	-968	2327	5156	107

Table S14. Hydrogen Atom Coordinates (Å×10^4) and Isotropic Displacement Parameters (Å^2×10^3) for **HKFI$_2$** (CCDC 2345911).

Atom	*x*	*y*	*z*	U(eq)
H3B	4470(60)	8350(30)	7080(30)	102
H1A	1497.81	4025.01	7294.65	71
H1B	10320.12	6641.9	10818.17	85
H2A	1024.19	5561.14	8215.65	74
H2B	7526.16	5897.94	10470.18	94
H3A	1515.46	7375.99	7441.02	68
H3BA	5882.52	6758.36	9121.44	81
H10A	2931.4	7605.71	3609.98	68
H10B	8849.03	10413.63	7319.56	74
H11A	3522.74	7013.34	2030.01	79
H11B	10877.98	11885	6981.9	83
H12A	3759.91	5108.1	1843.07	86
H12B	13539.42	11897.85	7851.87	91
H13A	3323.07	3737.4	3224.4	78
H13B	14155.63	10459.97	9173.02	87

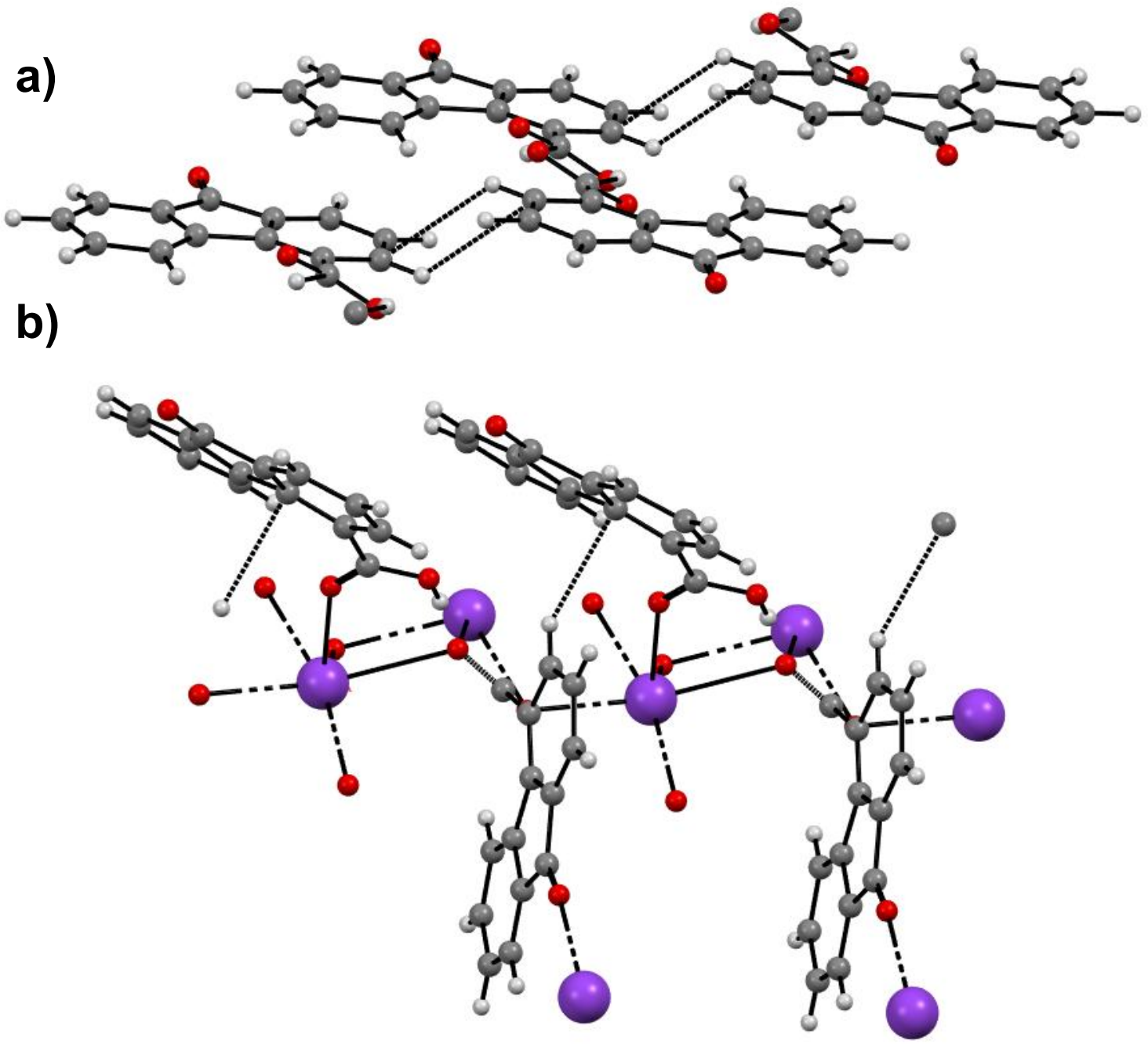

Fig. S7. a) CH-π interaction in HFI. b) CH-π interaction in $HKFI_2$ (CCDC 2345911).

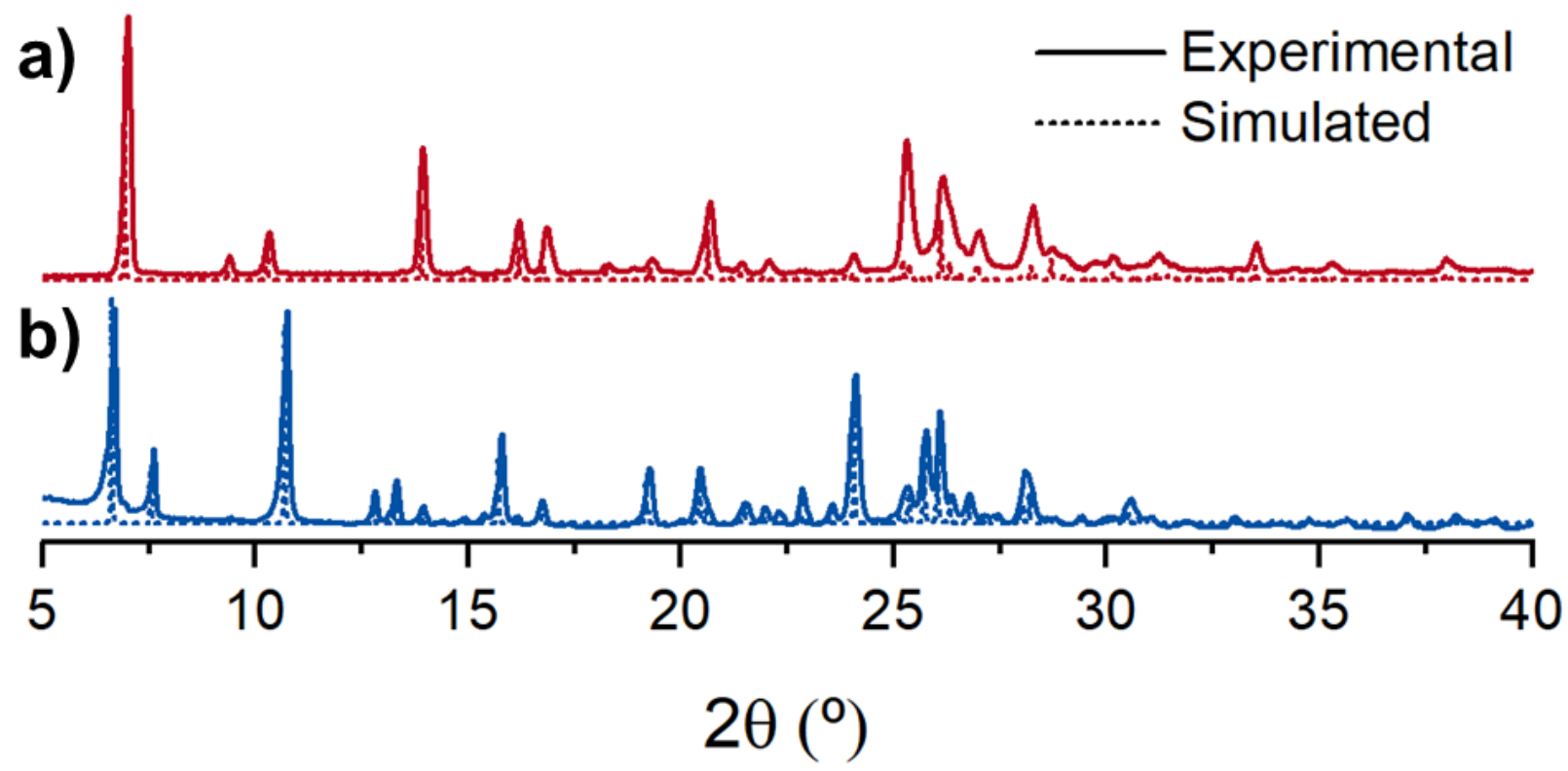

Fig. S8. Experimental and simulated PXRD patterns of a) $HKFI_2$ (CCDC 2345911) and (b) KFI (CCDC 2380849). The simulated patterns were generated from the single crystal structures. The good agreement between experimental and simulated data confirms that the bulk material corresponds to the respective phase.

5. Electrochemical performance

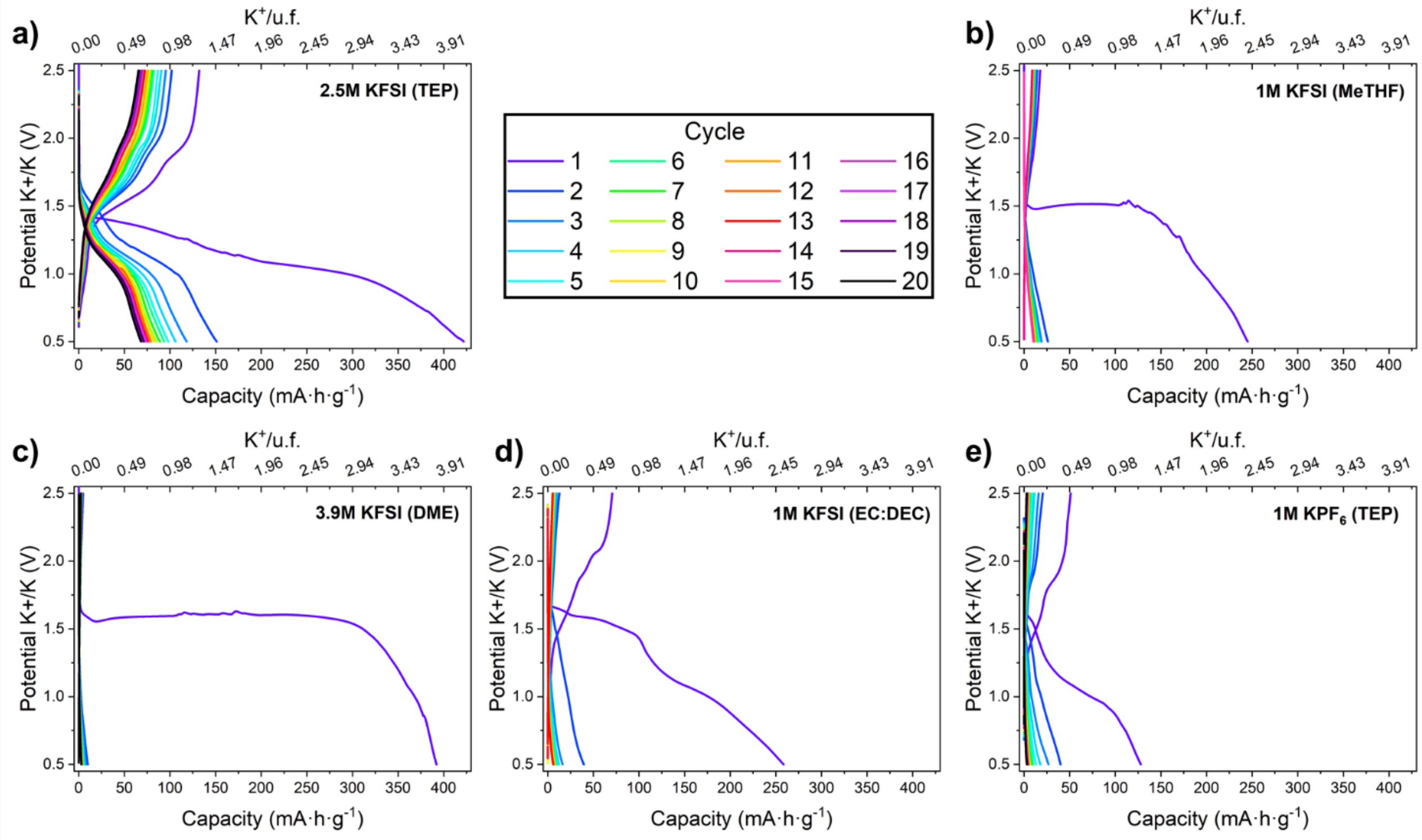

Fig. S9. GCD profiles of KFI at C/10 in different electrolytes: (a) 2.5 M KFSI in TEP, (b) 3.9 M KFSI in DME, (c) 1 M KFSI in EC:DEC (1:1), (d) 1 M KFSI in MeTHF, and (e) 1 M KPF_6 in TEP.

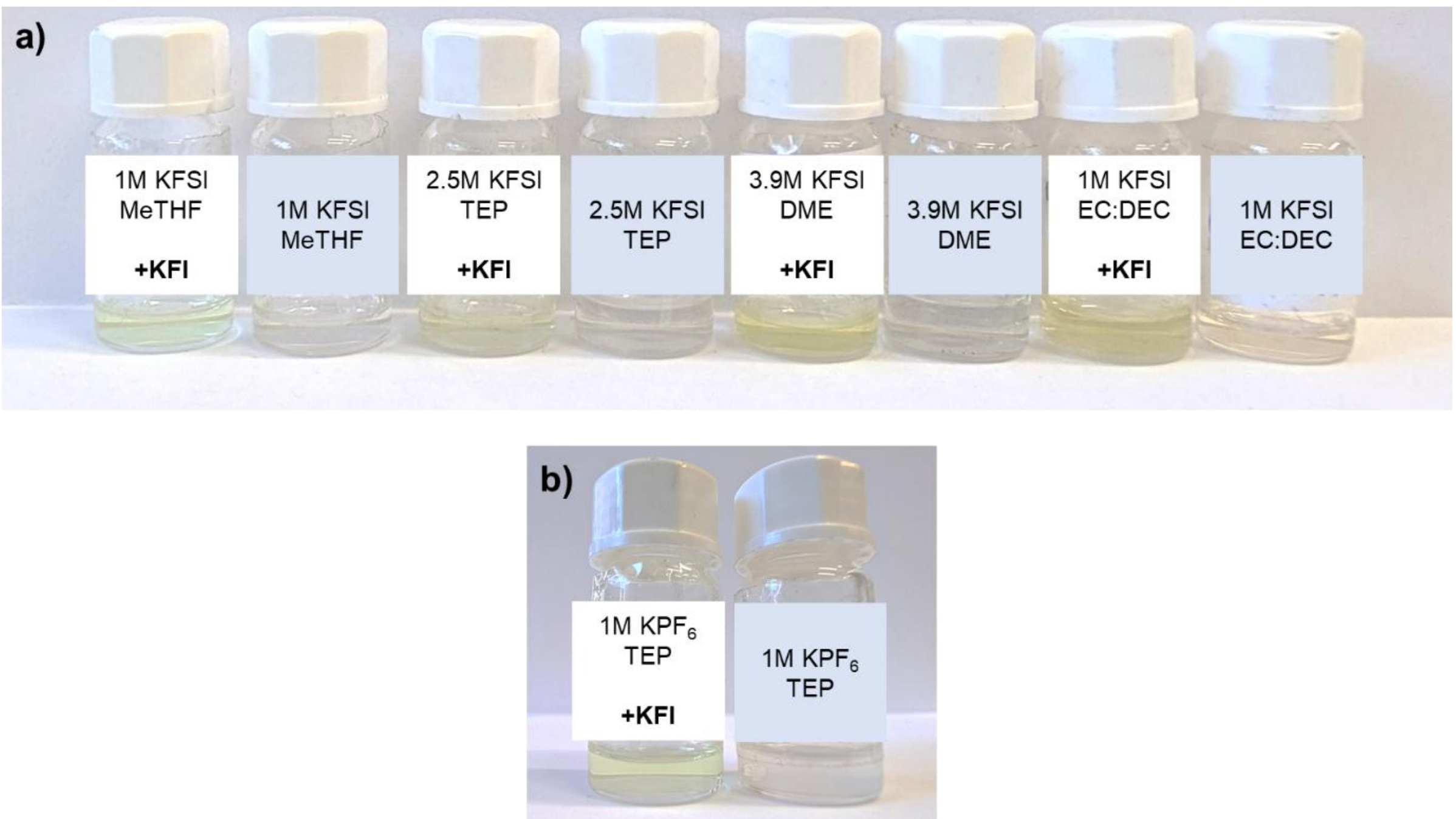

Fig. S10. Photographs of KFI after 48 h of contact with different potassium electrolytes, together with the corresponding blank electrolytes without KFI: (a) KFSI-based electrolytes and (b) 1 M KPF_6/TEP

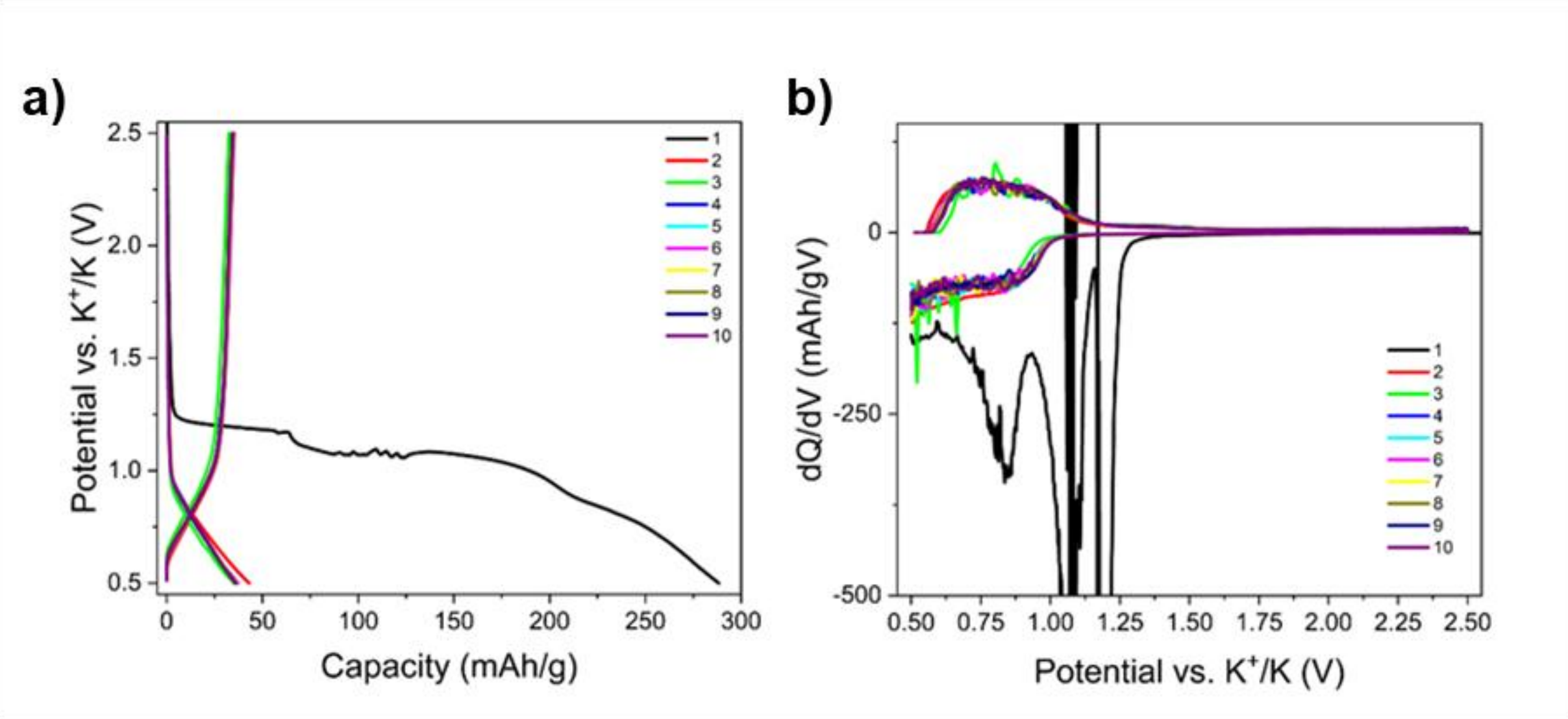

Fig. S11. Electrochemical behaviour of C65 in potassium half-cells using 2.5M KFSI in TEP as electrolyte and a current density of 7.2 mA/g: (a) voltage-capacity profile and (b) dQ/dV plot.

The applied current was calculated following the same approach used for the KFI electrodes by considering the theoretical active-material mass that would correspond to an equivalent electrode containing 70 wt.% KFI and 30 wt.% C65. This current corresponds to 7.2 mA/g when normalized to the actual mass of C65 in the blank electrode. For data analysis, the measured capacities were also normalized with respect to the actual mass of C65.

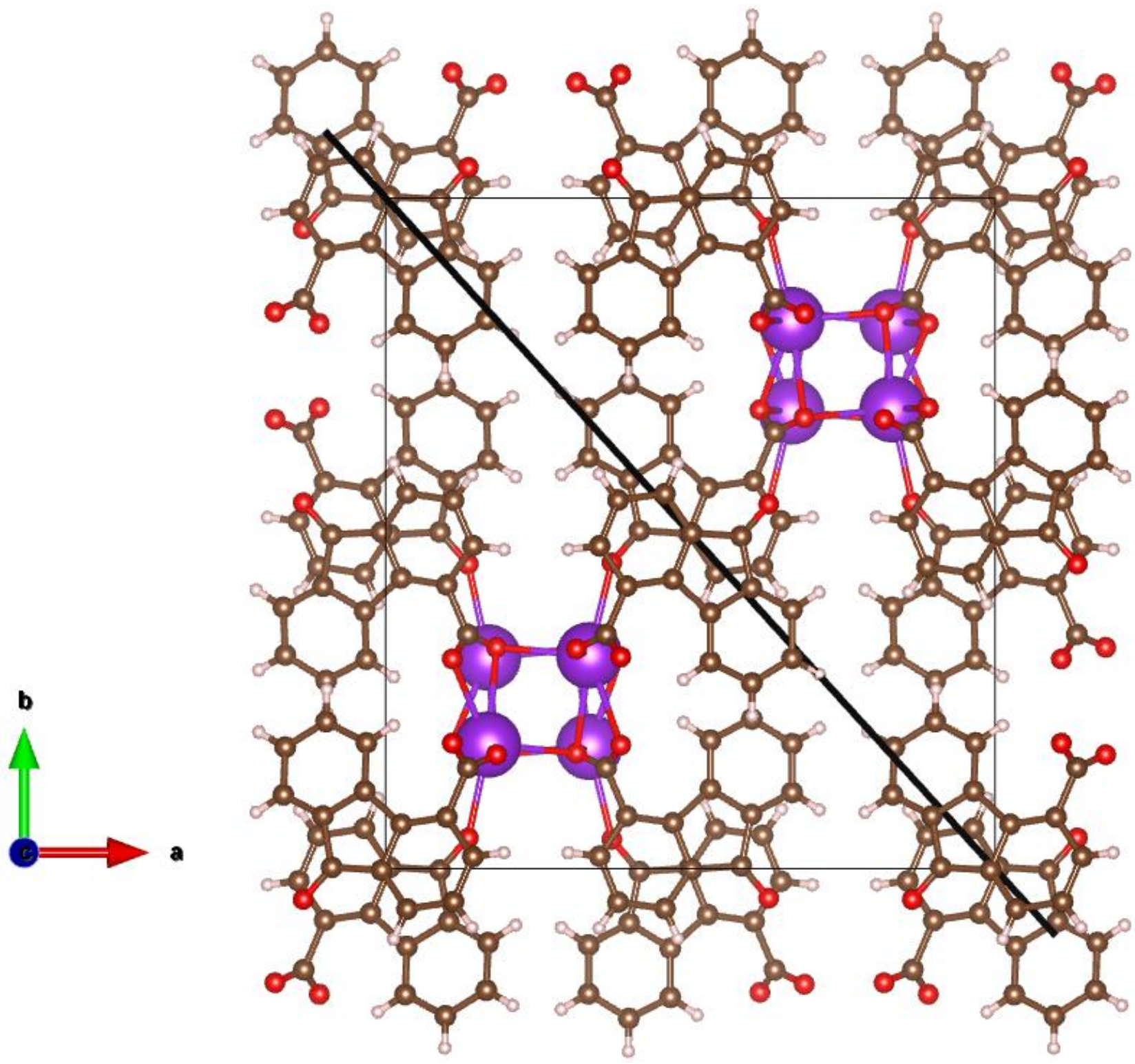

Fig. S12. View of the KFI crystal structure showing a representative member of the (110) family of lattice planes.

6. Toxicity

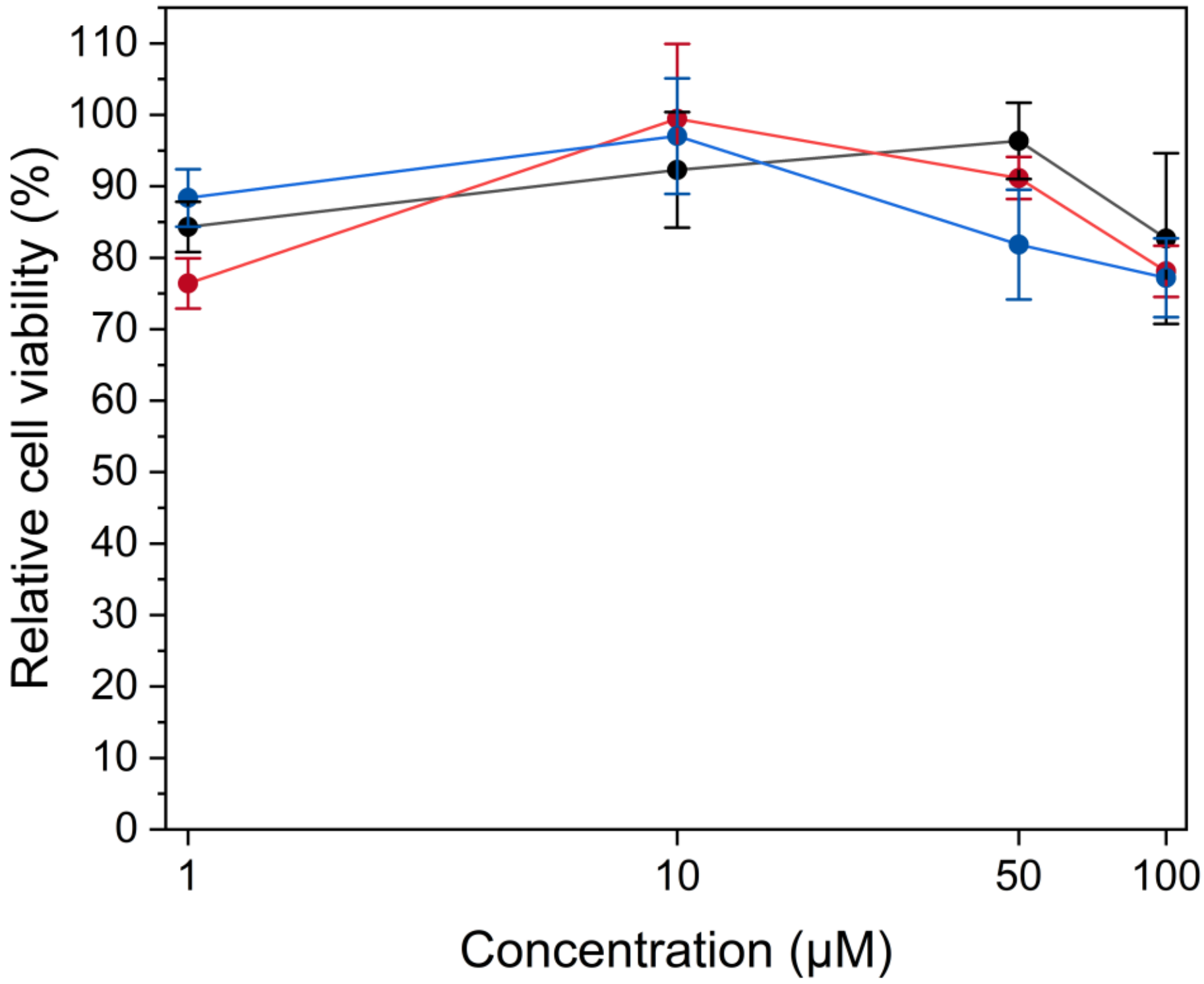

Fig. S13. MTT-based cytotoxicity assessment of HFI, $HKFI_2$ and KFI on human dermal fibroblasts (HDFn) after 24 h incubation. Error bars represent SD (n = 3).
